# Using chemical and DNA marker analysis to authenticate a high-value food, manuka honey

**DOI:** 10.1038/s41538-018-0016-6

**Published:** 2018-05-22

**Authors:** Claire M. McDonald, Suzanne E. Keeling, Mark J. Brewer, Steve C. Hathaway

**Affiliations:** 1 Ministry for Primary Industries, Wellington, New Zealand; 2 Biomathematics and Statistics Scotland, Aberdeen, UK

**Keywords:** Ecology, Natural products

## Abstract

Ensuring the authenticity of food is a rapidly emerging issue, especially in regard to high-value products that are marketed through increasingly complex global food chains. With the ever-increasing potential for mislabeling, fraud and adulteration, governments are increasingly having to invest in, and assure, the authenticity of foods in international trade. This is particularly the case for manuka honey, an iconic New Zealand food product. We show how the authenticity of a specific type of honey can be determined using a combination of chemicals derived from nectar and DNA derived from pollen. We employ an inter-disciplinary approach to evaluate a selection of authenticity markers, followed by classification modelling to produce criteria that consistently identify manuka honey from New Zealand. The outcome of our work provides robust identification criteria that can be applied in a regulatory setting to authenticate a high-value natural food. Our approach can transfer to other foods where assurance of authenticity must take into account a high level of natural variability.

## Food authentication: combining markers to help verify a specialty honey

High value speciality food products are threatened by mislabeling, fraud, and adulteration, which calls for practical authentication measures. Research led by the Ministry for Primary Industries in New Zealand was used to develop an analytical strategy that combines four chemicals and a DNA marker to authenticate mānuka honey, a speciality export from New Zealand. They found that a combination of chemicals from the mānuka plants nectar and species-specific DNA from mānuka pollen could be used as authenticity markers, when tested against a variety of plant and honey samples. Based on quantitative measurements of the markers, they used a classification modelling method to establish the identification criteria for both monofloral and multifloral mānuka honey. Combining multiple marker data and statistical classification analysis may also help develop other food authentication measures.

## Introduction

Ensuring food authenticity is a global issue. Recent examples of fraudulent activity include the addition of melamine to milk products,^1^ horse meat substitution,^2^ and seafood mislabelling.^3^ Manuka honey, a premium New Zealand export, is also a target for misrepresentation and there have been a number of claims in the international marketplace of mislabelling. In the case of honey which is always derived from multiple plant sources, addressing such claims is difficult, especially since honey composition varies from location to location and season to season. Currently, there is no regulatory standard for authentication of manuka honey from New Zealand and label claims are based on industry-agreed grading systems. The development of a robust, scientifically defensible standard for provision of government-to-government assurances of authenticity has now become a regulatory imperative.

In New Zealand, manuka honey is sourced from *Leptospermum scoparium* J. R. Forst & G. Forst, 1776, the dominant *Leptospermum* species. A member of the Myrtaceae family, it is one of over 70 species in the *Leptospermum* genus found in several countries including New Zealand and Australia.^4^

Currently, there are several industry approaches to identifying and marketing monofloral manuka honey, i.e., honey sourced predominantly from *L. scoparium*. The main approach is based on methylglyoxal, a chemical associated with antibacterial activity when medical grade honey is applied topically.^5,6^ Methylglyoxal is produced in the hive from the conversion of dihydroxyacetone, found in *L. scoparium* nectar and related species.^5,7^ However, methylglyoxal is highly problematic for product authenticity as it is not unique to manuka honey and levels increase and then decrease over time.^8,9^ More recently, leptosperin has been used by industry to support identification. However, it is readily found in Australian *Leptospermum*-type honeys typically at higher concentrations than in New Zealand,^10,11^ and so requires further assessment.

Monofloral manuka honey is also marketed by industry as containing at least 70% manuka pollen grains. The 70% threshold arose from a study suggesting that manuka honey should predominantly be composed of *Leptospermum* pollen.^12^ However, this study had a small sample size (*n* = 6) and relied on a test method with poor specificity due to microscopic pollen counts also including morphologically similar pollen from kanuka (*Kunzea ericoides*), a closely related Myrtaceae species.

Developing effective identification criteria for a specified floral honey type requires closely linking the source plant with the honey. With manuka honey, ideally markers would only be found from *L. scoparium* in New Zealand. However, this is unlikely as all honey types will have contributions from other plant species near the apiary site and *L. scoparium* grows in other countries. Therefore, it is important to not only consider the target plant species, *L. scoparium*, but also related *Leptospermum* species and other plant species involved in honey production in New Zealand. Another important consideration is the use of more than one marker to identify manuka honey to minimise the likelihood of fraud or adulteration, hence a multifaceted approach would be beneficial.^13,14^

In this study, we identified potential markers by (1) building on previously published studies (Table 1) and (2) specific development of DNA markers from pollen. Chemical markers associated with either *L. scoparium* or manuka honey in the science literature were selected. While our focus was on *L. scoparium*-derived honey, we also included markers associated with kanuka honey, to enable better characterisation of the honey samples tested in the study and to assist with determining floral origin. *Kunzea ericoides* and *L. scoparium* were once thought to be the same species and can have overlapping flowering periods. Many beekeepers maintain that manuka honey can be derived from both species.

**Table 1:** Summary of chemical marker assessment as potential authenticity markers using plant and honey data from 2014/2015 season

Chemical marker	Only found in manuka plants	Separates^a^ manuka from other plant species	Separates^a^ manuka honey from other New Zealand honey types	Separates^a^ monofloral from multifloral manuka honey	Relatively^b^ stable over time and temperature	Limited^c^ regional trends
2′-methoxyacetophenone (2′-MAP)^1–3^	Yes	Yes	Yes	Yes	Yes	Yes
2-methoxybenzoic acid (2-MBA)^4^	No	Yes	Yes	Yes	Yes	Yes
4-hydroxyphenyllactic acid (4-HPA)^1^	No	Yes	Yes	Yes	Yes	Yes
3-phenyllactic acid (3-PA)^1,4,5^	No	Yes	Yes	No	Yes	Yes
Methyl syringate (MS)^2^	No	Yes	Yes	No	No	NA
Leptosperin^6^	No	Yes	Yes	No	No	NA
Dihydroxyacetone (DHA)^7^	No	Yes	Yes	No	No	NA
Methylglyoxal (MG)^7^	No	No	Yes	Yes	No	NA
Kojic acid (KA)^2^	No	No	Yes	No	NA	NA
4-methoxyphenyllactic acid (4-MPA)^2^	No	No	Yes	No	NA	NA
Lumichrome^2,8^	No	No	No	No	NA	NA

*Note*: NA—not applicable, not tested for or not assessed as these markers were not suitable to distinguish manuka plants and honey from other plants and honey

Honey types referred in the table reflect the *original identification* used by the supplier

^a^“Separates” refers to a statistically significant difference at alpha 0.05

^b^“Relatively” refers to either no statistically significant difference at alpha 0.05 between different time points and/or temperatures or no consistent change across honey samples

^c^“Limited” refers to no consistent variation across regions and/or between years

**Table 2:** Misclassification categories of CART model predictions by honey type

	

*Note*: Dark blue cells are irrelevant misclassifications, light blue cells are mild misclassifications, and orange cells are severe misclassifications. Grey cells are classifications which align with original supplier identification

We describe a systematic approach for producing identification criteria that demonstrate authenticity of manuka honey from New Zealand. Key aspects include: establishing reference collections of plant material and honeys; sample testing of plant nectar, pollen, and honey; assessing marker suitability; and application of classification modelling to produce identification criteria. This work sets a benchmark for other similar challenges for authenticating other foods and illustrates the importance of combining a number of scientific disciplines to solve a complex problem.

## Results

Authenticity markers were determined using a number of assessments; these informed which markers were selected for the CART (classification and regression tree). Initially, 11 chemical markers (Table 1) and 2 DNA markers were evaluated. Assessment of chemical marker suitability involved determining specificity and analysing concentrations in nectar and honey samples plus assessing stability, regional, and temporal variation. After a systematic evaluation process using marker data from plant and honey samples collected in 2014/2015, the set of markers was reduced to those most suitable. Selected markers were further assessed using samples collected in 2015/2016 and included in the CART (Table 1). As the DNA markers were derived from pollen, determining specificity with the source plant and presence in plants and honeys across all New Zealand regions was a key requirement.

### Marker specificity and suitability

#### DNA markers

**Fig. 1:**
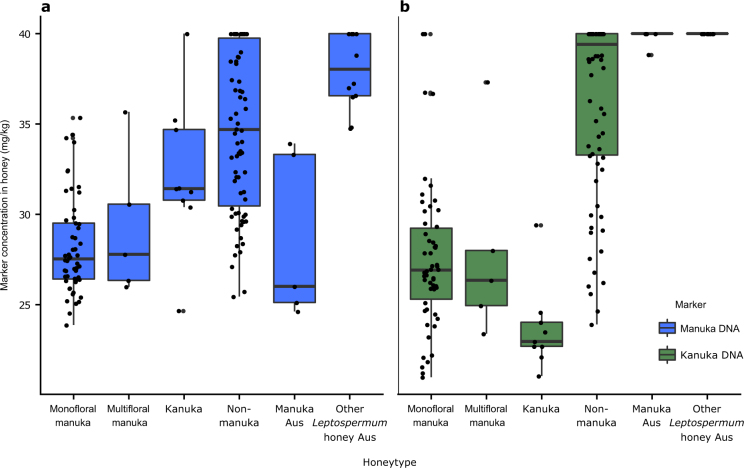
Concentration of DNA markers in each honey type collected in 2014/2015 from New Zealand and Australia. **a** Manuka DNA marker concentrations; **b** kanuka DNA marker concentrations. The distribution of the data is summarised in boxplots (the central line, median; box limits, first and third quartiles; whiskers, 1.5× inter-quartile range; points, outlier data beyond the end of the whiskers) and the concentration for individual samples are plotted. Honey types are from New Zealand unless they have “Aus” in their name in which case they are from Australia

Species-specific DNA markers for *L. scoparium* and *K. ericoides* were identified from samples collected across a range of habitats in New Zealand.^15^ Both DNA markers were detected in significantly different concentrations between the honey types collected from across New Zealand (Manuka DNA: LR test comparing model with honey type as explanatory variable and the null model, *χ*^2^_(1)_ = 112.84, *p* < 0.001; Kanuka DNA: LR test comparing model with honey type as explanatory variable and the null model, *χ*^2^_(1)_ = 143.34, *p* < 0.001), making them suitable for inclusion in CART modelling (Fig. 1). Further, the greater concentrations of the kanuka DNA marker in monofloral manuka honey compared with non-manuka honey (Tukey’s HSD test, mean difference = −0.28, *t*_(126)_ = 11.28, *p* < 0.001), Australian manuka honey (Tukey’s HSD test, mean difference = −0.38, *t*_(57)_ = 5.88, *p* < 0.001), and Other Leptospermum honey from Australia (Tukey’s HSD test, mean difference = −0.38, *t*_(64)_ = 8.75, *p* < 0.001) indicate the potential value for inclusion in a CART.

### Chemical markers

#### Nectar

**Fig. 2:**
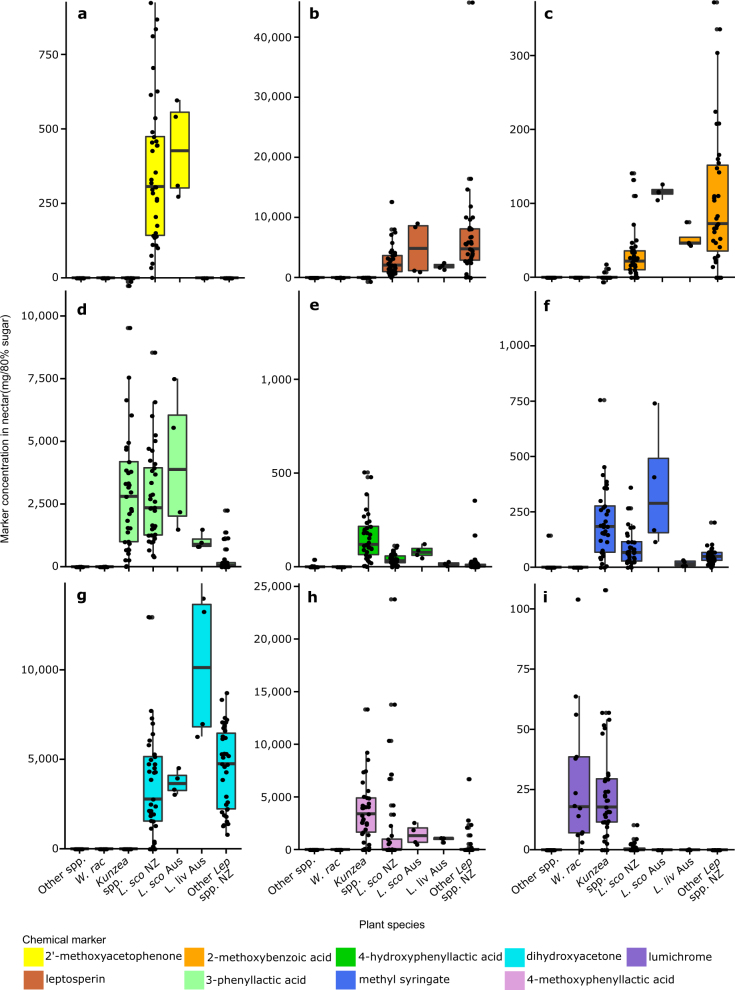
Concentration of chemical markers in the nectar of the plant species collected in 2014/2015 from New Zealand and Australia. Plant species have been abbreviated as follows: Other spp. (4 species from New Zealand); *W. rac* (*Weimannia racemosa*); *L. sco* NZ (*Leptospermum scoparium* from New Zealand); *L. sco* Aus (*L. scoparium* from Australia); *L. liv* Aus (*L. liversidgei* from Australia); and Other *Lep* spp. NZ (13 other *Leptospermum* species from New Zealand). **a** 2′-methoxyacetophenone; **b** leptosperin; **c** 2-methoxybenzoic acid; **d** 3-phenyllactic acid; **e** 4-hydroxyphenyllactic acid; **f** methyl syringate; **g** dihydroxyacetone; **h** 4-methoxyphenyllactic acid; **i** lumichrome. The distribution of the data is summarised in boxplots (the central line, median; box limits, first and third quartiles; whiskers, 1.5× inter-quartile range; points, outlier data beyond the end of the whiskers) and the concentration for individual samples are plotted

From the 2014/2015 nectar samples (New Zealand: *n* = 161, Australia *n* = 8), all chemical markers were found in more than one species except 2′-MAP which was only found in *L. scoparium* (Fig. 2a). DHA was restricted to *Leptospermum* spp. nectar (Fig. 2g). 2-MBA and 4-MPA were restricted to *Leptospermum* species and *Kunzea* spp. (Fig. 2c, h). The remaining chemical markers known to be present in nectar (leptosperin, 4-HPA, 3-PA, MS, and lumichrome) were detected in the nectar of *Leptospermum* spp., *Kunzea* spp., and other species (Fig. 2). Note: kojic acid and MG have not been reported in nectar to date. For chemical markers found in non-*Leptospermum* spp., significant concentration differences between plant species supported their potential use as authenticity markers.

Across all bootstrap simulations, 2-MBA concentration was significantly greater in *L. scoparium* than *Kunzea* spp., but in 98.2% of simulations the concentration was significantly less in *L. scoparium* from New Zealand compared to Australia (Supplementary Fig. 1b). The concentration of 4-MPA was significantly greater in *Kunzea* spp. than *L. scoparium*; however, only 40.5% of *L. scoparium* samples had detectable levels of 4-MPA (Supplementary Fig. 1g).

Leptosperin was detected in all *Leptospermum* spp. tested as well as *Weinmannia racemosa* and *Kunzea* spp., albeit at significantly lower concentrations than *Leptospermum* spp. (Fig. 2b and Supplementary Fig. 1a). 3-PA was detected in all species except for *Trifolium repens* and *Ixerba brexioides*. However, 3-PA was significantly greater in *L. scoparium* and *Kunzea* spp. than in the other New Zealand species tested, including those grouped under “other *Leptospermum* spp.” (Supplementary Fig. 1c). Similarly, 4-HPA was detected in the majority of species, except for *T. repens*, *I. brexioides*, and one sample of *Kn. excelsa* (Fig. 2e). 4-HPA concentrations in *L. scoparium* were significantly greater than *Metrosideros excelsa*, *Knightia excelsa*, and “other *Leptospermum* spp.” from New Zealand, but at significantly lower concentrations than *Kunzea* spp. and *L. scoparium* from Australia (Supplementary Fig. 1e). Methyl syringate was detected in all *Leptospermum* spp., *Kunzea* spp., and in one sample of *T. repens* (Fig. 2f). The concentration of MS in *L. scoparium* nectar was significantly greater than *L. liversidgei* (Australia), but was significantly different from both *K. ericoides* and *L. scoparium* (Australia) in 82.2 and 93.2% of simulations, respectively (Supplementary Fig. 1f).

Although lumichrome was detected in *W. racemosa*, *Kunzea* spp., *L. scoparium*, and *L. liversidgei* (Australia) (Fig. 2i), it was found in significantly greater concentrations in *Kunzea* spp. and *W. racemosa* than all other plant species (Supplementary Fig. 1h). The concentration of lumichrome in *L. scoparium* nectar was very low, being only significantly greater than *Kn. excelsa* and *T. repens* in 90.9 and 64% of simulations, respectively, despite lumichrome not being detected in either of these species above limit of reporting (LOR) (Supplementary Fig. 1h).

#### Honey

**Fig. 3:**
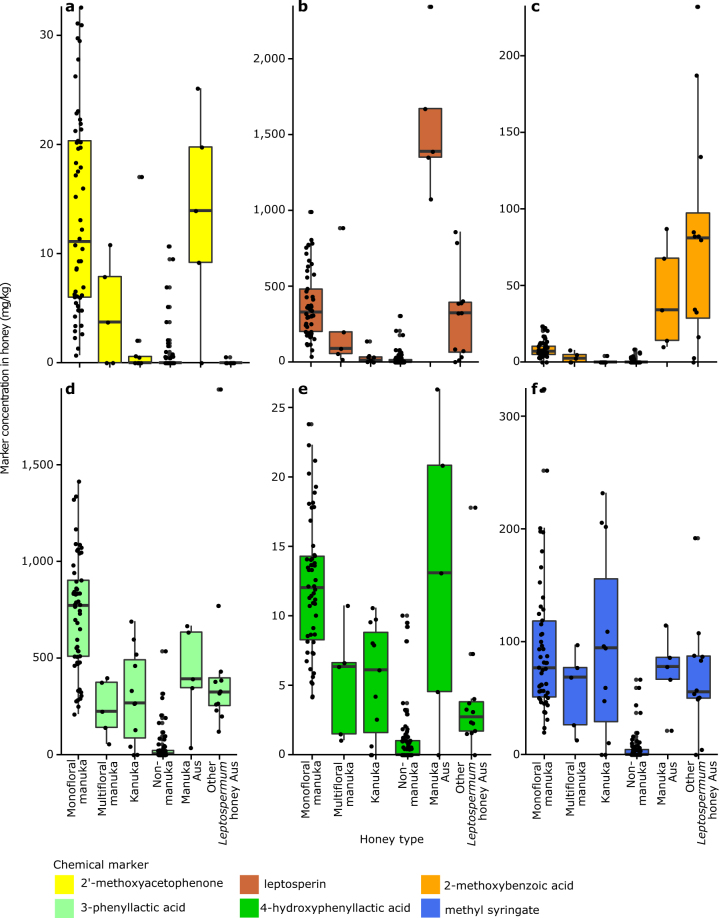
Concentration of six chemical markers in each honey type collected in 2014/2015 from New Zealand and Australia. **a** 2′-methoxyacetophenone; **b** leptosperin; **c** 2-methoxybenzoic acid; **d** 3-phenyllactic acid; **e** 4-hydroxyphenyllactic acid; **f** methyl syringate. The distribution of the data is summarised in boxplots (the central line, median; box limits, first and third quartiles, whiskers, 1.5× inter-quartile range; points, outlier data beyond the end of the whiskers) and the concentration for individual samples are plotted. Honey types are from New Zealand unless they have “Aus” in their name in which case they are from Australia

**Fig. 4:**
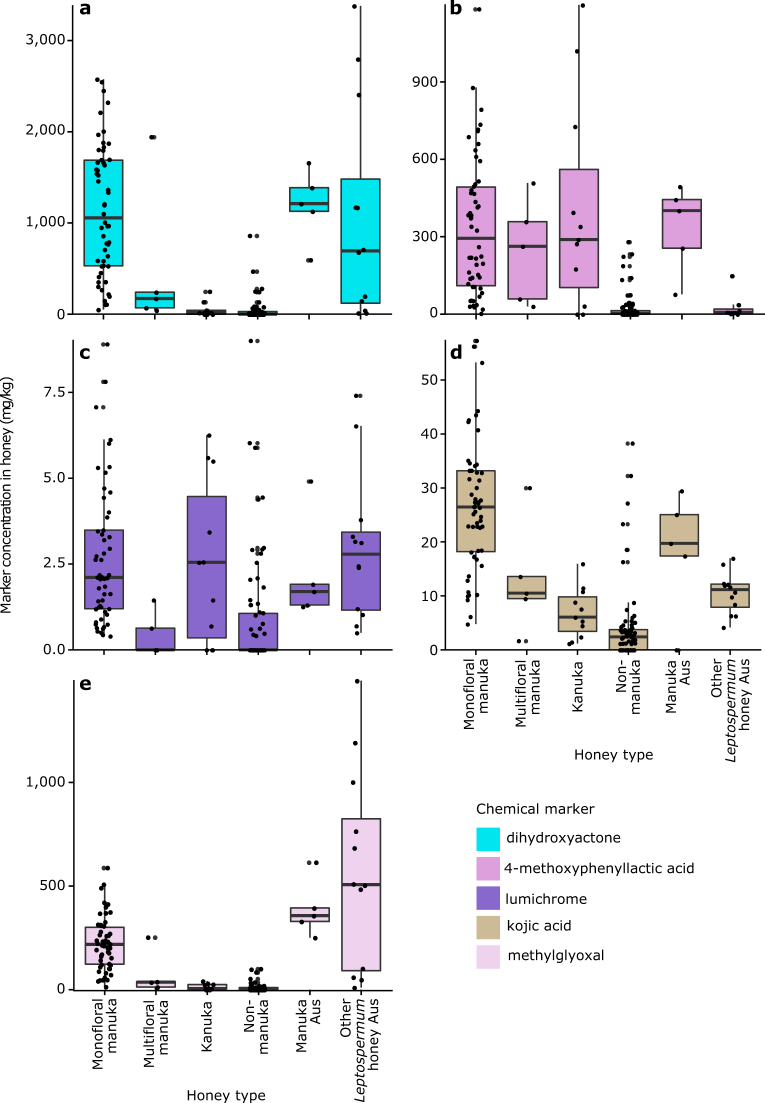
Concentration of five chemical markers in each honey type collected in 2014/2015 from New Zealand and Australia. **a** dihydroxyacetone; **b** 4-methoxyphenyllactic acid; **c** lumichrome; **d** kojic acid; **e** methylglyoxal. The distribution of the data is summarised in boxplots (the central line, median; box limits, first and third quartiles; whiskers, 1.5× inter-quartile range; points, outlier data beyond the end of the whiskers) and the concentration for individual samples are plotted. Honey types are from New Zealand unless they have “Aus” in their name in which case they are from Australia

Ideally, marker concentrations would be significantly greater in manuka honey enabling separation from other honey types. Chemical markers were detected in 2014/2015 honey samples with varying concentrations within and between standardised honey types (monofloral manuka (*n* = 54); multifloral manuka (*n* = 5); kanuka (*n* = 11); non-manuka (*n* = 75); manuka honey from Australia (*n* = 5); and *Leptospermum* honey from Australia (*n* = 15)) (Figs. 3, 4).

Concentrations of 2′-MAP, DHA, 2-MBA, 4-MPA, leptosperin, 3-PA, 4-HPA, MG, and kojic acid were significantly greater in monofloral manuka honey than kanuka and non-manuka honey (Supplementary Fig. 2). Significantly greater concentrations of 2′-MAP, 2-MBA, and MG were detected in monofloral manuka compared to multifloral manuka honey (Supplementary Fig. 2). When compared against manuka honey from Australia, most markers were in similar concentrations (Figs. 3, 4), except for leptosperin and 2-MBA. Both these markers were significantly lower in monofloral and multifloral manuka honey from New Zealand than in manuka honey from Australia (Supplementary Fig. 2b, c).

Methyl syringate concentrations were significantly greater in monofloral manuka than non-manuka honey, but not significantly different from the other four honey types (Supplementary Fig. 2g). Lumichrome was detected in low concentrations in all monofloral manuka, manuka from Australia, kanuka, and non-manuka honeys (Fig. 4).

Specificity and suitability assessment indicated that eight chemical markers found in *L. scoparium* nectar and their concentrations in honey have the potential for separating manuka honey from other honey types. These markers include: 2′-MAP, DHA, 2-MBA, leptosperin, 3-PA, 4-HPA, MS, and MG and were subsequently assessed for stability.

### Stability

Laboratory experiments informed marker stability following exposure to three different temperatures over a short time period. Markers that showed a significant change, particularly in a downwards direction over time, would not be ideal authenticity markers. Experiments showed that 2′-MAP and 4-HPA concentrations did not show a significant change with either time or temperature (Supplementary Table 1). 3-PA concentrations showed a small but significant decrease with increasing temperature (*F*-test, *F*_(3,15)_ = 5.81, *p* = 0.008) but not time. The concentration of 2-MBA changed with temperature (*F*-test, *F*_(3,15)_ = 3.87, *p* = 0.03), but this was only significant between samples stored at 20 and 35 °C where the concentration increased after an initial decrease (Supplementary Table 2). In contrast, a significant change in concentration with both temperature and time was observed for MS, leptosperin, DHA, and MG (Supplementary Tables 1–3). The results from the stability experiments indicated 2′-MAP, 2-MBA, 3-PA, and 4-HPA were relatively stable over time and were subsequently assessed for regional and temporal variation.

### Regional and temporal variation of markers in manuka

A suitable honey authenticity marker should be detected in all New Zealand regions where the plant grows and manuka honey is produced. We did not find consistent large-scale spatial trends across markers in *L. scoparium* nectar or manuka honey, but as expected there were some significant regional differences for some markers (Supplementary Table 4). Differences were not consistent between flowering seasons, e.g., significant regional differences were found in 3-PA nectar concentrations for 2014/2015 (*F*-test, *n* = 37, *F*_(6, 30)_ = 12.23, *p* < 0.001), but not for 2015/2016 (*F*-test, *n* = 115, *F*_(11, 103)_ = 0.53, *p* = 0.876). However, 3-PA in manuka honey did differ with region and production year (Supplementary Table 4). The concentrations of 2′-MAP, 4-HPA, and 2-MBA in the nectar did not differ with region in either flowering season, but significant differences between regions were found in the honey (Supplementary Table 4). Where significant regional differences were observed for a marker, the same regions did not differ consistently for each marker.

Our assessment of temporal differences for the four markers found significant differences in mean levels in manuka honey between the two honey production years, except for 2-MBA (Supplementary Table 5). In general, the concentrations of each marker were significantly greater in manuka honey produced in 2014/2015 than 2015/2016 (Supplementary Fig. 3). CART analysis allows these differences to be accommodated.

### Developing identification criteria

Using six markers (2′-MAP, 2-MBA, 3-PA, 4-HPA, manuka DNA, and kanuka DNA) baseline CARTs were fitted using data (2014/2015 and 2015/2016) from the following standardised honey types: monofloral manuka (*n* = 173), multifloral manuka (*n* = 70), kanuka (*n* = 29), and non-manuka honey types (e.g., clover; *n* = 219). Honey samples from Australia (*n* = 44) and other countries (*n* = 71) were also used to fit CARTs; archive samples from 2009/2010 to 2014/2015 (*n* = 169) were used to test CARTs. Differences in marker concentration between honey production years supported fitting and testing CARTs with separate production years and highlighted the importance of collecting multiple years’ data. We used a classification matrix to define a classification outcome as severe, mild, or irrelevant (Table 2).

### Baseline CART from 2014/2015 data

The CART from the 2014/2015 training data included 3-PA, 2-MBA, 4-HPA, manuka, and kanuka DNA as markers to separate the honey types (Supplementary Fig. 4). The first tree split was 3-PA at 54.60 mg/kg for 3-PA, with higher values indicating kanuka, and monofloral manuka as identified by the supplier. Lower 3-PA threshold values indicated non-manuka, Australian, or honey from other countries. There were two pathways to identify multifloral manuka honey, both higher and lower than the 3-PA threshold value.

The overall within-sample classification rate based on supplier identification was 68%. In comparison, the out-of-sample classification rates using both 2015/2016 archive data as test sets was 55% in both cases. However, misclassifications of test data tended to be of less severe forms: for the 2015/2016 test set, samples misclassified were split into irrelevant/mild/severe as 19/26/1%; the equivalent for the archive samples was 38/7/1%.

### Baseline CART from 2015/2016 data

The CART with the 2015/2016 training data is more complex (Supplementary Fig. 4), having eleven rather than seven splits (Supplementary Fig. 5). As before, the first split was 3-PA, but with a higher threshold (99.48 mg/kg). This tree also contained the markers 2-MBA, 4-HPA, manuka DNA, and kanuka DNA.

The overall within-sample correct classification rate based on supplier identification was 62%. In comparison, the out-of-sample correct classification rates using 2014/2015 and archive data as test sets was 29 and 42%, respectively; much lower than above. Again, misclassifications for test data were mostly less severe forms; 40/28/4% for 2014/2015 data and 49/9/1% for archive data.

### False positives and false negatives for monofloral manuka honey

Overall false positive (OFP) and false negative rates (OFN) for monofloral manuka honey were generally higher for out-of-sample assessments using 2015/2016 data as the training set (2014/2015: OFP = 0.50, OFN = 0.83; archive: OFP = 0.14, OFN = 0.43) than those using 2014/2015 data (2015/2016: OFP = 0.32, OFN = 0.13; archive: OFP = 0.21, OFN = 0.18). Even for a within-sample test, CART fitted on 2015/2016 data results in misclassification of 9% of samples as “severe false negatives” (SFN), resulting in supplier identified monofloral manuka honey being classified as non-manuka. The out-of-sample severe false positives were low for all scenarios (<2%), but the out-of-sample SFN for 2014/2015 training data were lower (2015/2016: SFN = 0.01) than using 2015/2016 data (2014/2015: SFN = 0.06).

### Sensitivity assessment

CART outcomes were not highly sensitive to the changes explored through the bootstrap simulations. Further detail can be found in Supplementary Figs. 6–10 and Supplementary Tables 6–11; key results for out-of-sample assessment are:

- 3-PA was selected by CART fitting in nearly all bootstrap simulations, confirming its importance to a definition;
- out-of-sample assessment of CART differed with honey production year used as the training data set, although the differences were not significant (Supplementary Fig. 6 and Supplementary Table 6);
- similar out-of-sample classifications and severe misclassifications between honey production areas, but significantly greater classification of monofloral manuka honey using CARTs built with North Island data (Supplementary Fig. 7 and Supplementary Table 7);
- out-of-sample classifications were significantly lower when a 4-factor honey classification variable was used over a 6-factor classification (Supplementary Figs. 6a, 8a); however, each marker was selected in the CARTs in a similar number of bootstrap simulations (Supplementary Table 6);
- out-of-sample classifications for monofloral manuka honey and the number of severe misclassifications did not significantly change when using fewer chemical markers (Supplementary Figs. 6, 10);
- effects of changing LOR values were negligible (Supplementary Tables 9 and 10).

### Establishing and testing the robustness of identification criteria

The baseline CARTs and results from sensitivity analyses were combined to create a final set of candidate models, with refinements to minimise the risk of overfitting. The 2014/2015 training data produced the best out-of-sample classification rules, so this was selected as the training set for fitting subsequent CARTs.

Sensitivity analyses indicated that the number of markers as an input to CART was unimportant, particularly for classification of monofloral manuka honey. Hence, from the six markers used in the previous CARTs, only 3-PA and both DNA markers were used. The number of honey classes did influence results, therefore honey type as both a 4-level and 6-level response variable were considered. This approach was chosen for refining initial CARTs as it gave more control over the process than the standard method of “pruning” trees produced by CART. For example, “severe” misclassifications would not be penalised more heavily if the standard method was used.

**Fig. 5:**
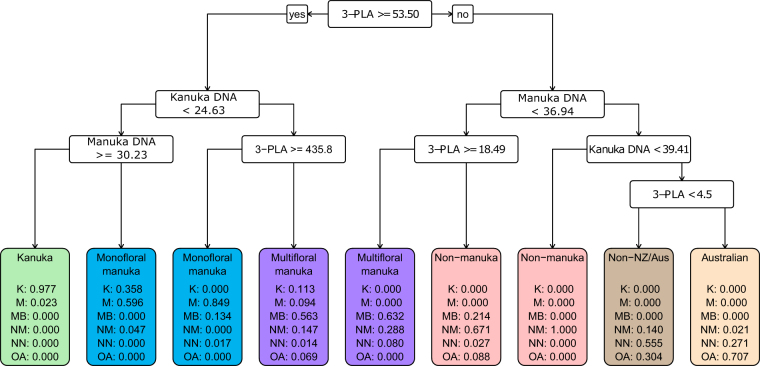
CART built using 2014/2015 data as training set and honey type as a 6-level response variable. Markers used to build the CART included 3-phenyllactic acid (3-PLA); manuka DNA and kanuka DNA. At each split point in the tree if the condition is met the path furthest left is chosen and if not then the path furthest right is chosen. The predicted class proportions at each terminal child node are shown. Class abbreviations are as follows: Kanuka honey (K); Monofloral manuka (M); Multifloral manuka (MB); Non-manuka (NM); Non-NZ/Aus (NN); and Australian (AU). Units for chemical markers are mg/kg. Units for DNA markers are C_q_

**Fig. 6:**
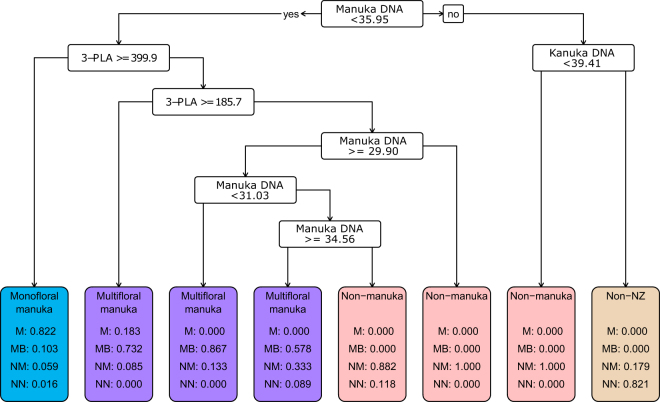
CART built using 2014/2015 data as training set and honey type as a 4-level response variable. Markers used to build the CART included 3-phenyllactic acid (3-PLA); manuka DNA and kanuka DNA. At each split point in the tree if the condition is met the path furthest left is chosen and if not then the path furthest right is chosen. The predicted class proportions at each terminal child node are shown. Class abbreviations are as follows: Monofloral manuka (M); Multifloral manuka (MB); Non-manuka (NM); Non-NZ (NN). Units for chemical markers are mg/kg. Units for DNA markers are C_q_

Via this process, we obtained two CARTs for classifying monofloral and multifloral manuka honey (Figs. 5, 6). The CART for the 6-level honey type classification provided two conditional pathways for identifying monofloral manuka honey and another two for multifloral manuka honey (Fig. 5). The CART for the 4-level honey type classification had only one pathway for monofloral and three for multifloral manuka honey (Fig. 6).

Both the baseline CART and sensitivity analyses selected 2-MBA and 4-HPA in a fair proportion of simulations. Therefore, thresholds provided in the baseline CARTs (Supplementary Figs. 4, 5) were included in options for the candidate identification criteria. Threshold levels for 2-MBA and 4-HPA were ≥LOR so the presence or absence of these chemical markers was added to the options; criteria without 2-MBA and 4-HPA classified more honey samples from Australia and other countries as multifloral manuka honey, with one sample classified as monofloral manuka honey. Therefore, to minimise severe misclassifications, both 2-MBA and 4-HPA should be included in the candidate identification criteria (Table 3: Options 1 and 2).

**Table 3:** Options of identification criteria for monofloral and multifloral manuka honey based on results of CART model outputs applied to the 2014/2015 data set

	Option 1^a^				Option 2^b^		Option 3^a^				Option 4^b^	
	Monofloral		Multifloral		Monofloral	Multifloral	Monofloral		Multifloral		Monofloral	Multifloral
Marker	A	B	A	B	A	A	A	B	A	B	A	A
3-PA	≥50	≥400	≥50 and <400	≥20 and <50	≥400	<400	≥50	≥400	≥50 and <400	≥20 and <50	≥400	<400
2′-MAP	NA	NA	NA	NA	NA	NA	≥1	≥1	≥1	≥1	≥1	≥1
2-MBA	≥1	≥1	≥1	≥1	≥1	≥1	≥1	≥1	≥1	≥1	≥1	≥1
4-HPA	≥1	≥1	≥1	≥1	≥1	≥1	≥1	≥1	≥1	≥1	≥1	≥1
Manuka DNA	<30	<36	<36	<36	<36	<36	<30	<36	<36	<36	<36	<36
Kanuka DNA	<30	<25	>25	NA	NA	NA	<25	≥25	≥25	NA	NA	NA

Units for chemicals are mg/kg. Units for DNA are C_q_. Values for 3-PA have been rounded

^a^A 6-level honey type response variable produced pathways, A and B, either can predict the given honey type

^b^A 4-level honey type response variable produced one pathway for each honey type

Marker level thresholds in each identification criteria were rounded after CART analysis for ultimate ease of implementation. When the exact marker values from CART were applied to Option 1 (e.g., 435 mg/kg instead of 400 mg/kg and 18.5 mg/kg instead of 20 mg/kg for 3-PA) the impact was minimal. For the 2014/2015 data, no samples were reclassified, and only 2.6 and 1.8% of New Zealand honey samples were reclassified for the 2015/2016 data and archive data, respectively. The use of rounded values is therefore clearly justified on the evidence from available data.

A further two options were tested and considered (Table 3: Options 3 and 4). These simply added 2′-MAP to the previous options. Although not selected as a variable in the baseline CART for either training data set, in the sensitivity analyses 2′-MAP was included in many trees from the bootstrap simulations. Adding 2′-MAP reclassified a small number of monofloral and multifloral manuka honey samples as non-manuka relative to Options 1 and 2, but fewer non-manuka samples were classified as multifloral manuka honey. In comparison to Options 1 and 3, Options 2 and 4 led to fewer monofloral manuka honey samples being classified as non-manuka honey. Options 1 and 3 (Table 3) were discounted as the pathways were too complex, and this would be challenging to apply within a regulatory setting. By contrast, the identification criteria of Options 2 and 4 (Table 3) were relatively simple, with 2′-MAP in Option 4 being the only difference.

Systematic assessment of bias (plus/minus 5, 10, 15, and 20%) for both chemical and DNA markers in Option 4 showed that there was minimal effect on sample reclassification for all test data for chemicals (Supplementary Table 11). However, systematic bias of DNA marker values had minimal effect on sample reclassification at lower levels of systematic bias, but at an upward bias of 20% sample reclassification was higher (Supplementary Table 12). Importantly, no samples from Australia or other countries were reclassified in the systematic bias assessment.

In comparison to Options 2 and 4, Options 1 and 3 had a minimal level of 3-PA of 20 mg/kg level (specifically 18.17 mg/kg) for multifloral manuka honey providing additional from non-manuka samples. Sensitivity analyses results also supported using 3-PA to separate these honey types. Setting a lower limit of 20 mg/kg will prohibit a honey that tests below LOR being labelled as monofloral or multifloral manuka honey.

After assessing all four options, the following markers at specified levels were considered in combination to form identification criteria to authenticate manuka honey (either monofloral or multifloral):

- ≥1 mg/kg for 2′-MAP, 2-MBA, and 4-HPA;
- ≥20 mg/kg 3-PA; and
- DNA from manuka pollen (<C_q_ 36 equivalent of 3.2 fg/µL DNA).

To further separate honey as either monofloral or multifloral manuka honey, 3-PA is required:

- Monofloral manuka honey = ≥ 400 mg/kg 3-PA,
- Multifloral manuka honey = ≥ 20 but <400 mg/kg 3-PA.

When the identification criteria were applied, 74% of supplier-identified monofloral manuka samples were classified as monofloral manuka and 56% of supplier-identified multifloral manuka samples were reclassified as monofloral manuka honey (Table 4). For those classified as multifloral manuka, 23% were supplier-identified as multifloral manuka. The remaining samples were originally labelled as monofloral manuka, kanuka, or non-manuka (Table 4).

**Table 4:** Comparison of original supplier identifications of honey samples against the criteria

		Identification using criteria		
Supplier identification	No. of samples	Not manuka (%)	Multifloral manuka (%)	Monofloral manuka (%)
Monofloral manuka	273	14	12	74
Multifloral manuka	95	21	23	56
Kanuka	30	60	17	23
Non-manuka (e.g., clover)	262	88	12	<1
Australian and other countries honey	115	100	0	0

## Discussion

Authentication of food products subject to natural variation is a considerable scientific challenge. Our study was complex; not only were we developing identification criteria suitable for regulatory use, we also had no “gold standard” for manuka honey with which to compare against. Also, the absence of an internationally agreed approach to what constitutes monofloral or multifloral honey for a particular honey type further added to the challenge. Thus, we used a systematic inter-disciplinary approach coupled with appropriate data analysis to authenticate manuka honey.

A key component of the study was selecting markers that were suitable for demonstrating authenticity. Ideally, markers would only be found in the source plant and not subject to any external influences (natural or artificial). As this was highly unlikely, we considered marker specificity, suitability, stability, and assessing regional and temporal influences to inform marker selection. As the DNA markers provided greater specificity to the source plant compared to chemical markers, we used different criteria when assessing their suitability. Chemical marker assessment involved evaluation of several parameters to inform marker selection for CART analysis.

Two markers (2′-MAP and manuka DNA) were specific to *L. scoparium* plants based on the samples that we tested. Specificity of other chemical markers were similar to that found in other studies, except for 2-MBA, leptosperin, and lumichrome. Other published work has shown these to be present in *L. scoparium* or members of the *Leptospermum* genus (2-MBA, leptosperin)^16,17^ and *K. ericoides* (lumichrome).^17^ However, we detected 2-MBA in *K. ericoides* and other *Leptospermum* species, leptosperin in *W. racemosa* and *K. ericoides* and lumichrome was detected in *K. ericoides*, *L. scoparium* (New Zealand), *L. liversidgei* (Australia), and *W. racemosa*. Finding differences between published studies is not surprising as we tested a larger sample set representing a greater diversity of plant species enabling wider specificity assessment.

As we did not want to rely on a single marker to identify manuka honey for use in a regulatory setting, we pursued a combination of markers from the outset in an effort to minimise opportunities for adulteration. As the presence of chemical markers alone was not effective for distinguishing different honeys, chemical marker suitability was further assessed by comparing nectar and honey concentrations. Markers and their concentrations that enabled distinction between plant species and/or separate honey types were considered candidate markers. Concentrations of markers to separate monofloral from multifloral manuka honey and to assist in supporting New Zealand floral origin were also assessed. We identified 2′-MAP, 2-MBA, 3-PA, 4-HPA, and confirmed that they were not unduly influenced by regional and temporal effects and were relatively stable. These four chemical markers and both DNA markers were established as candidates for evaluation using CART. The kanuka DNA marker was included as the honey data indicated that it was useful for separating key honey types and assisting with floral origin.

Although CART has not been used extensively in food authenticity studies, its use in other areas is relevant to developing identification criteria for manuka honey. CART has helped solve a number of classification problems in public health,^18–20^ ecology,^21,22^ and climate effects.^23^ In the food area, CART has determined regional or geographical origin of wine^24^ and olive oils.^25^ CART produces a set of clear and simple rules which is essential for understanding and implementation in a regulatory setting.

The proposed identification criteria were applied to all honey samples in our study, representing New Zealand manuka honey production areas, multiple suppliers, several production years, and overseas honeys. We observed that due to variation in classification success, especially between production years, it was important to develop, fit, and test models so that identification criteria represented honey from several different years. Fitting models to a combined data set would have increased misclassification rates, thus reducing the ability to differentiate between honey types.

Application of the proposed identification criteria to honey samples showed good agreement with original supplier identification. Agreement would always be somewhat less than 100% for several reasons, e.g., bias in supplier knowledge and experience, applying different industry approaches to identification, variability in bee foraging behaviour at the same apiary site or between seasons. Of interest was the classification of 56% of supplier-identified multifloral manuka as monofloral manuka honey. In these cases there was a greater contribution from *L. scoparium* than thought by the supplier. Archive sample results showed that the identification criteria were valid as far back as 2009/2010, suggesting the suitable markers can be detected over time periods corresponding to product shelf life. However, as honey is a natural product, the identification criteria should be monitored for changes due to climate change or bee and plant disease events.

It is also important to recognise that unlike traditional approaches which use a percentage estimate to imply contribution from both nectar and pollen, our identification criteria do not allow this. Establishing a percentage to identify a honey type attributed to the source plant is complex, requiring a full understanding of ecological interactions at the apiary site. For example, the number of trees in flower, and the expression and composition of nectar by those flowers, is influenced by climatic and microclimatic effects.^26^ In addition, nectar volume collected by the bees is influenced by competition with other nectarivores, bee preferences for foraging from the source plant and the distance they are willing to fly from the hive to forage.^27^ All these factors make it difficult to estimate with any confidence or accuracy the proportion of the nectar in a honey. As such, our identification criteria cannot be used to determine a percentage of the manuka plant in the honey. However, based on the evidence produced, we can be confident that markers and associated levels can consistently demonstrate authenticity and distinction from other floral sources.

Demonstrating the authenticity of food as labelled by the producer is of key importance to regulators, consumers, and industry. Any identification criteria used to provide food assurances must be supported by defendable and transparent scientific evidence that also meets the requirements for use in regulation. It is also important that markers can be readily and reliably tested for in commercial laboratories worldwide. Both the chemical and DNA markers can be readily tested for using standard methods and instrumentation available at most commercial testing laboratories. Future adaptations may be required to address changes in industry practices, advancements in technology, and to manage any new threats from illegal practices.

We have defined a honey type in a way that is transparent, systematic, and scientifically robust. Application of readily available analytical techniques and sophisticated data analyses required multidisciplinary knowledge of ecology, biological systems, laboratory test methods, and applied statistics. Statistical classification analysis has been shown to be a highly appropriate tool in this context. Our work provides a robust platform for tackling food authenticity issues of global importance affecting international trade.

## Methods

A number of chemical and DNA markers were evaluated for use in the identification and authentication of manuka honey from New Zealand (Table 1). Plant and honey samples from reference collections primarily sourced from New Zealand were tested for the markers. After initial assessment of specificity and suitability, candidate markers were identified. Candidate markers were further assessed following testing of a larger number of both plant and honey samples. The quantitative values of the markers were analysed using CARTs^28^ to produce identification criteria for both monofloral and multifloral manuka honey from New Zealand.

### Sample collection

#### Plants

Our sampling plan targeted the collection of *L. scoparium*, other *Leptospermum* species (New Zealand and Australia), *Kunzea ericoides*, and other plant species commonly used in New Zealand honey production. Plant samples were collected from New Zealand (29 species; Supplementary Table 13) and from Australia (5 *Leptospermum* species; Supplementary Table 13) during two flowering seasons (2014/2015 and 2015/2016). Herbarium specimens for all plants collected in New Zealand were deposited in the National Forestry Herbarium (NZFRI), New Zealand.

Nectar was collected from New Zealand (12 regions) and Australia (5 states) using a modified technique.^29^ Ten flowers from each plant were flushed with a total of 100 µL of deionised water using a micropipette. Nectar collection for plant species with smaller flowers used a stereo microscope. Nectar was chilled in the field and then stored at −80 °C. A total of 506 samples were used for testing, while the remaining nectars were archived.

Specific sites within regions were targeted for sample collection using the known distribution of each plant species rather than random selection. Information recorded for each collection included: GPS coordinates, altitude, collector, time, and weather conditions. During the second flowering season, *L. scoparium* plants were sampled, where present, from five habitat types within each New Zealand region: calcareous soil, coastal sand/headland, dry ridge, low-altitude bog, and montane areas.

#### Honey

We sourced honey samples from single apiaries in New Zealand over 7 production years from 121 suppliers. A single apiary site is a geographic location at which honey was harvested from one or more hives prior to honey being blended with other honey. Samples were obtained directly from beekeepers and honey packers. We targeted collection of honey immediately post-harvest for 2014/2015 and 2015/2016 (*n* = 491), whereas honey samples (*n* = 169) from previous production years (2009–2014) were sourced from industry archives. We also sourced honey from 16 countries (*n* = 135) directly from beekeepers where possible, but mostly from retail products. We tested and analysed data from 795 honeys in total (Supplementary Table 14).

New Zealand suppliers provided traceability information including the likely main floral source, apiary site location, flowering period, harvest date, and time and temperature of storage. Traceability data was standardised to ensure consistency in format and descriptive terminology used. Honey samples sourced from other countries had limited traceability information.

### Sample testing

Chemical markers in both nectar and honey samples were measured using liquid chromatography diode array (UV/Vis) detection (DHA and MG) or using liquid chromatography tandem mass spectrometry (all other chemicals).^30^ Sugars in nectar were measured using Ultra (-High) Performance Liquid Chromatography Fluorescence detection/Diode Array Detection. The LOR for all test methods was 1 mg/kg for honey and 0.01 mg/L for nectar.

DNA markers in honey samples were detected using the ManKan^TM^ qPCR multiplex^15^ which independently detects DNA of *L. scoparium* (manuka) and *K. ericoides* (kanuka). Results are reported as a quantification cycle number (C_q_ value), with 36 being LOR.

### Marker evaluation

A key aspect of this study was determining the specificity and suitability of markers in relation to *L. scoparium*. DNA marker specificity was determined by assessing the ability of the marker to detect the target plant species but not related species or other species involved in New Zealand honey production.^15^ Specimens from existing herbarium collections (National Forestry Herbarium, New Zealand; Auckland War Memorial Museum Herbarium, New Zealand; and the Western Australian Herbarium, Australia) were also included.

Chemical markers were assessed by evaluating both presence/absence and concentration. For example, a chemical marker found in more than one plant species may still be suitable if the concentration enabled separation from other plant species. We evaluated variability in chemical marker concentration in both nectar and honey (2014/2015 and 2015/2016) based on temporal and regional differences.

### NaNNaNStability experiments

To help inform marker selection, a short stability trial was conducted. If the values of a chemical marker changed significantly after a short incubation time, particularly in a downwards direction, it would not be a suitable marker given the long shelf life of honey (3 years or more). Chemical marker stability was evaluated using six honey manuka samples obtained from several different suppliers. Homogenised honey samples were sub-sampled in triplicate for testing at time zero and at a second time point for three temperatures (4, 20, and 35 °C). After storage (*t* = 68 days), sub-samples were homogenised and concentrations of each marker determined. Archive honey samples were used to assess DNA stability as well as long-term stability of chemical markers as this is more reflective of industry practice.

### NaNNaNStatistical analyses

To ensure samples could be appropriately grouped and compared, nectar and honey data were standardised prior to analysis (Supplementary Table 14). For nectar data, chemical concentrations were converted into the mg/80% sugar scale and tested for collector bias prior to data analyses (data not shown). Unless otherwise stated, the concentration data for each chemical marker was log-transformed (using natural logs) prior to model fitting. DNA marker data were used directly with no truncation or standardisation of values above the LOR. The data from each collection year were analysed separately for individual marker evaluation unless stated otherwise. All analyses were performed using statistical software R v3.2.0.^31^

#### Marker evaluation analyses

The differences in the mean C_q_ value for each DNA marker were compared by fitting an ANOVA model to the data and using honey type as a fixed effect. We used Tukey HSD multiple comparisons of means and *p*-values were adjusted using the Benjamini and Hochberg method (R package multcomp). The variance in C_q_ values for the manuka DNA marker was dependent on honey type, therefore a variance structure which differed per honey type was included in the model (R package nlme).

Using an ANOVA approach, we tested for differences between levels of chemical markers in different species of plant or honey types. As a number of test results were less than the LOR, a simulation approach was used to reduce bias. Simulated values were drawn in two ways depending on the distribution of the chemicals in the nectar/honey type:

1. If a nectar/honey type had some values below and above the LOR, then a random number was sampled from a uniform distribution for each value below the LOR in the range [0, LOR].
2. When all values of a nectar/honey type were below the LOR, then a value was simulated from a beta distribution with shape parameters 1 and 40 (ensuring consistent left skew of values) and scaled according to the LOR by multiplication.

The second simulation method was important for analysing nectar marker data, as it avoided problems associated with the necessary scaling by sugar content. We simulated 1000 new data sets for each marker comparison based on the 2014/2015 data and fitted an ANOVA model for each set. We used Tukey HSD multiple comparisons of means and *p*-values were adjusted using the Benjamini and Hochberg method (R package multcomp^32^). Predicted mean values were estimated using a winsorised variance to account for large variances in simulations (R package psych v1.6.9^33^). Results are summarised by the proportion of simulations when two nectar/honey types were significantly different at alpha level of 0.05, the mean difference between the two groups, and the mean error of this difference.

#### Variation in *L. scoparium* nectar with habitat type

We used linear models with Gaussian errors to test for the effect of habitat type on the concentration of markers in *L. scoparium* nectar from the 2015/2016 data. In all models, data for each chemical marker was the response variable (sugar scale equivalent) in turn and habitat type was used as the single explanatory variable (5-level factor). Marker values below the LOR were set to 0.1 mg/L prior to conversion to sugar scale equivalent. Results are presented for 2′-MAP, 2-MBA, 3-PA, 4-HPA, and DHA. The number of values below the LOR was relatively low, with the exception of DHA. Therefore, the effect of habitat type was tested using the simulation approach detailed for the marker evaluation assessment.

#### Stability experiments data analysis

Differences in concentrations of each chemical marker at each temperature, and between the two time periods, were examined using an ANOVA approach. The response variable was the concentration value of each chemical marker and two models were fitted with either the explanatory variables of sample number (6-level factor) and storage temperature (4-level factor) or explanatory variables of sample number and testing date (2-level factor). A step-wise model selection procedure investigated the significance of the interaction between sample number and storage temperature or sample number and testing data. When testing for statistical significance the mean value for each specific sample was used.

#### Regional and temporal variation of markers in manuka

We explored spatial variation in chemical markers in manuka nectar and honey by fitting a linear model on the concentrations and using regional classification as an explanatory variable (12-level factor). For temporal variation we focussed on manuka honey and compared distributions of the chemical markers in different years by fitting a linear model using year as the explanatory variable (3-level factor: 2 honey production years and archive samples).

#### Developing the identification criteria

CART is a non-parametric statistical method used for explaining and predicting categorical and continuous response variables.^28^ We used CART to produce simple rules for classifying honey samples. In fitting CARTs, we used the “gini” index as the impurity function, a uniform prior for prior class proportions with other starting parameters the default for “rpart” function from R package rpart v4.1–9.^34^ As the differences in sample size between honey types classification would influence the classification, we did not use class proportions estimated from the data as priors.

We used “honey type” as the response variable; the explanatory variables were the proposed authenticity markers obtained after specificity and suitability assessment. With the exception of Australia, *Leptospermum* are absent or uncommon in the overseas countries from which samples were collected, hence these samples were grouped into one single level named “non-NZ/Aus”. Australian samples were considered separately due to *Leptospermum* species being native. Hence, honey type was represented by six classes: monofloral manuka; multifloral manuka; kanuka; non-manuka; Australian; and non-NZ/Aus honey.

Given observed temporal variation in markers across the sampling years, we split the data into two parts, one for each honey production year (2014/2015 and 2015/2016), using each in turn as the training set to build the model. So that all honey types in the data could be classified, we included honey samples from Australia and non-NZ/Aus in the training set in addition to both honey production years. Archive samples were used as a test data set only.

Our aim was to use the CART method to produce a set of “baseline” models which would provide starting points for more detailed examination of the data. The baseline CART would not distinguish between different types of misclassification between honey types, and we are mainly interested in classifications involving manuka honey (and less in so-called “irrelevant misclassifications”—see the next section). The idea then was to refine and simplify the baseline CARTs, in order to produce candidate straightforward definitions for manuka honey, without harming classification success rates where it mattered.

In order to test the robustness of CART applied to our data, we employed both cross-validation and bootstrapping. We fitted the model 1000 times using bootstrap resampled versions of our training data set in order to assess the robustness of fitted classification trees; in particular, we were interested in how often each marker was included in a fitted tree, whether as first split or otherwise.

#### Assessing CART model output

The performance of the classification model was examined specifically for the honey samples labelled “monofloral manuka”. This was assessed by considering false positives and false negatives; false positives are the misclassification of other honey types as monofloral manuka honey, and false negatives are the misclassification of monofloral manuka honey as other honey types. Ideally, the selected model should minimise both of these misclassifications.

With no reference standards for identifying any of the honey types, and the likely variability associated with the labelling of the honey samples by suppliers, it is important to recognise that classification success rates may not be an accurate reflection of the actual performance of the classification model. For example, a honey sample labelled “multifloral manuka” by the supplier may be classed as a “monofloral manuka” because it is more similar to other monofloral manuka samples than the multifloral manuka samples. Also, the differing industry views on which plant species are represented in manuka and kanuka honey will influence classification success rates. To assess misclassifications, they were categorised as an ordered degree of misclassification: irrelevant, mild, or severe (Table 2); this allowed for a more robust comparison of fitted models.

Irrelevant misclassifications were not a priority outcome (e.g., misclassifying a non-NZ/Aus honey as an Australian honey was considered irrelevant for our purposes). Mild misclassifications were considered understandable and less crucial. For example, “kanuka” or “monofloral manuka” honey could fairly easily be classified as a “multifloral manuka” honey as the honey may have higher and lower quantities of manuka markers than the supplier originally thought. Similarly, “non-manuka” honey might be classified as “multifloral manuka” honey as manuka markers are likely to be present given the widespread distribution of *L. scoparium* throughout New Zealand. Severe misclassifications involved honey types classified as a different group, but where this could not be easily explained or would provide an inadequate identification criterion for manuka honey. For example, “non-manuka” honey classified as “monofloral manuka” honey was considered a severe misclassification.

#### Sensitivity assessment

The sensitivity of CART outputs to changes in the training data was examined under a range of scenarios:

1. Different honey production years: 2014/2015 vs. 2015/2016.
2. Different production areas: North Island of New Zealand vs. South Island of New Zealand.
3. Number of different honey types: Six classes as described earlier vs. four classes—“monofloral manuka”, “multifloral manuka”, “non-manuka”, and “non-New Zealand including Australian”.
4. Number of different markers used as explanatory variables: Results from the above scenarios were used to select the combination of markers tested. Samples from 1 honey production year was used as the training set, while the other production year and archive samples were used as test sets.

For each scenario, bootstrap sampling with replacement was used to subset the New Zealand samples. Data were resampled within each honey type; resampling was also performed separately on the samples from Australia and the non-NZ/Aus honey. The new bootstrapped data for the New Zealand, Australia, and non-NZ/Aus samples were used to refit the CART. This process was repeated 1000 times for each scenario and the bootstrap distribution obtained for each summary statistic that was used for the baseline scenario. A mean and 95% confidence interval from the bootstrap distributions were also calculated.

The procedure for LOR (as detailed in the marker evaluation) was then performed for all chemical markers selected for the CART. The original DNA data were also added to the new simulated chemical data to fit a new CART model. The effect of using simulated LOR values in the data set was assessed by comparing the classifications and misclassifications with those from CARTs fitted using the original chemical data. If correct classifications are higher and/or misclassifications are lower with the simulated chemical data, then it would be important to use outputs from CARTs built using simulated LOR values. Similarly to the bootstrap analysis, the simulation was repeated 1000 times for each scenario and the scenario tested on CARTs built with the data from each honey production year and tested on the other year’s data and the archive data set.

Similar to the process used for the chemical markers, DNA data values of C_q_ above 36 (the LOR) were recoded as 40, maximum assay value. We assessed this recoding effect by fitting a model with simulated values and comparing classification success with one using the original DNA data. Chemical markers were also included in CART and the same scenarios explored using training and test data from different honey production years.

#### Establishing and testing the robustness of identification criteria

CART results were used to evaluate which honey production year and which combination of markers would best provide the final CART(s). Rules from the CART(s) provided draft sets of identification criteria for both monofloral and multifloral manuka honey. Classification success of each set was examined and robustness tested by assessing classification when: (1) rounded versions of the split point thresholds were used and (2) systematic bias associated with laboratory testing were applied at levels of 5, 10, and 20%, as both overestimation and underestimation. We considered these two issues to aid understanding and implementation of the identification criteria. We assessed the proposed identification criteria by summarising the classification of the combined data for all honey samples collected and the regional differences in the classification of monofloral manuka.

### Data availability

All data used in this study are available by request to the Ministry for Primary Industries. To protect the confidentiality of honey suppliers and land owners, information that identifies the specific geographic location of the plant or honey samples will be provided in coded format.

## Electronic supplementary material

Supplementary Information

## Electronic supplementary material

**Supplementary information** accompanies the paper on the *npj Science of Food* website (10.1038/s41538-018-0016-6).
