# Supplementary material for: Using chemical and DNA marker analysis to authenticate a high-value food, manuka honey
Source: NPJ Sci Food. 2018 May 22;2:9. doi: 10.1038/s41538-018-0016-6 (PMC6550171; doi:10.1038/s41538-018-0016-6)
Supplement: Supplementary file 1 — Supplementary Information [file 41538_2018_16_MOESM1_ESM.pdf]

Supplementary Information

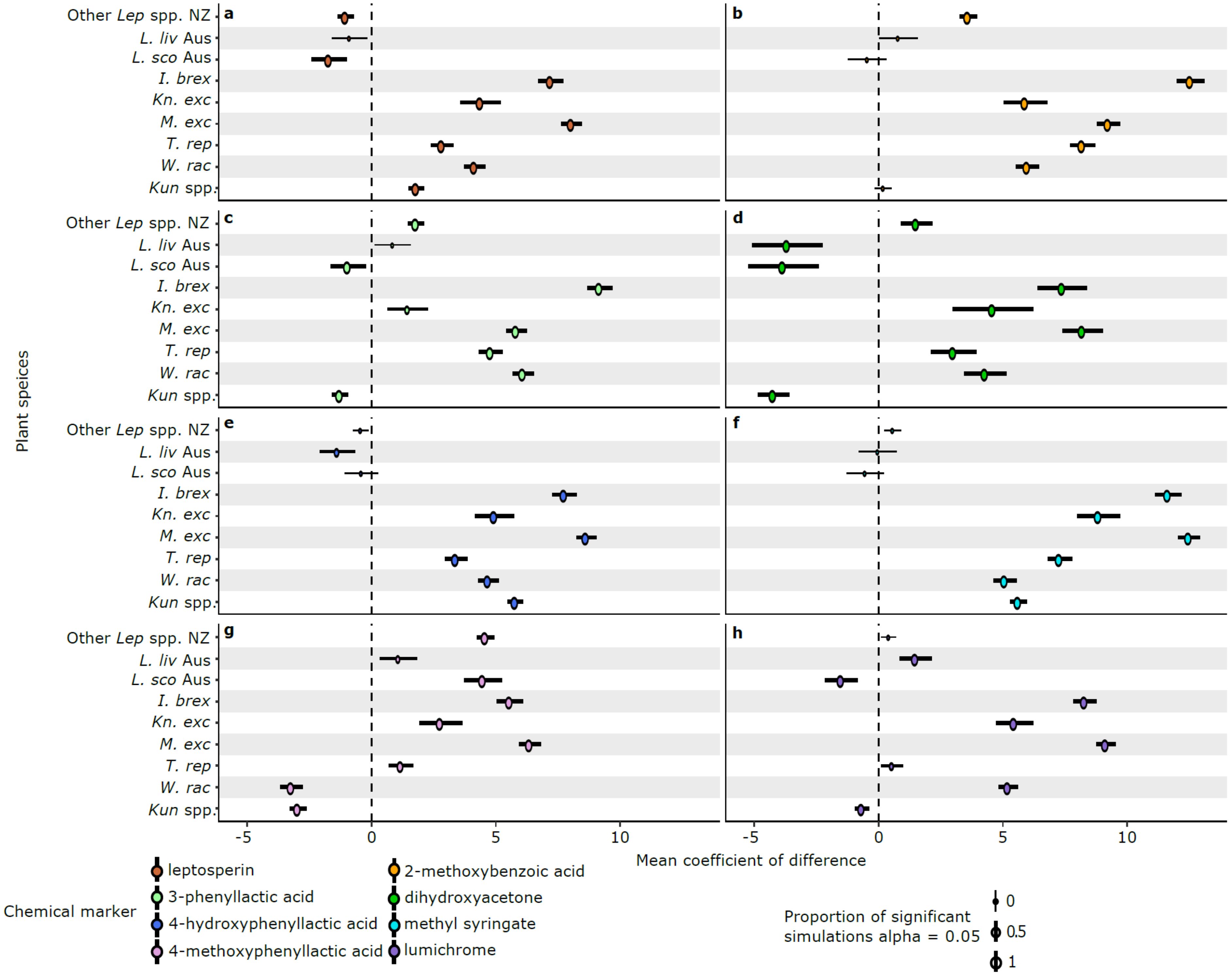

Supplementary Fig. 1 Bootstrap simulation results for concentration differences of each marker between *Leptospermum scoparium* and the other plant species collected in 2014/15 from New Zealand and Australia. Error bars show the mean coefficient of difference ± 1 standard error of difference from the bootstrap simulations. Plant species abbreviations are: Other *Lep* spp. NZ (Other *Leptospermum* spp. New Zealand), *L. liv* Aus (*L. liversidgei* Australia), *L. sco* Aus (*L. scoparium* Australia), *I. brex* (*Ixerba brexioides*), *Kn. exc* (*Knightia excelsa*), *M. exc* (*Metrosideros excelsa*), *T. rep* (*Trifolium repens*), W. rac (*Weimannia racemosa*) and *Kun* spp. (*Kunzea* spp.). (**a**) leptosperin; (**b**) 2-methoxybenzoic acid (2-MBA); (**c**) 3-phenyllactic acid (3-PA); (**d**) dihydroxyacetone; (**e**) 4-hydroxyphenyllactic acid (4-HPA); (**f**) methyl syringate; (**g**) 4-methoxyphenyllactic acid; (**h**) lumichrome.

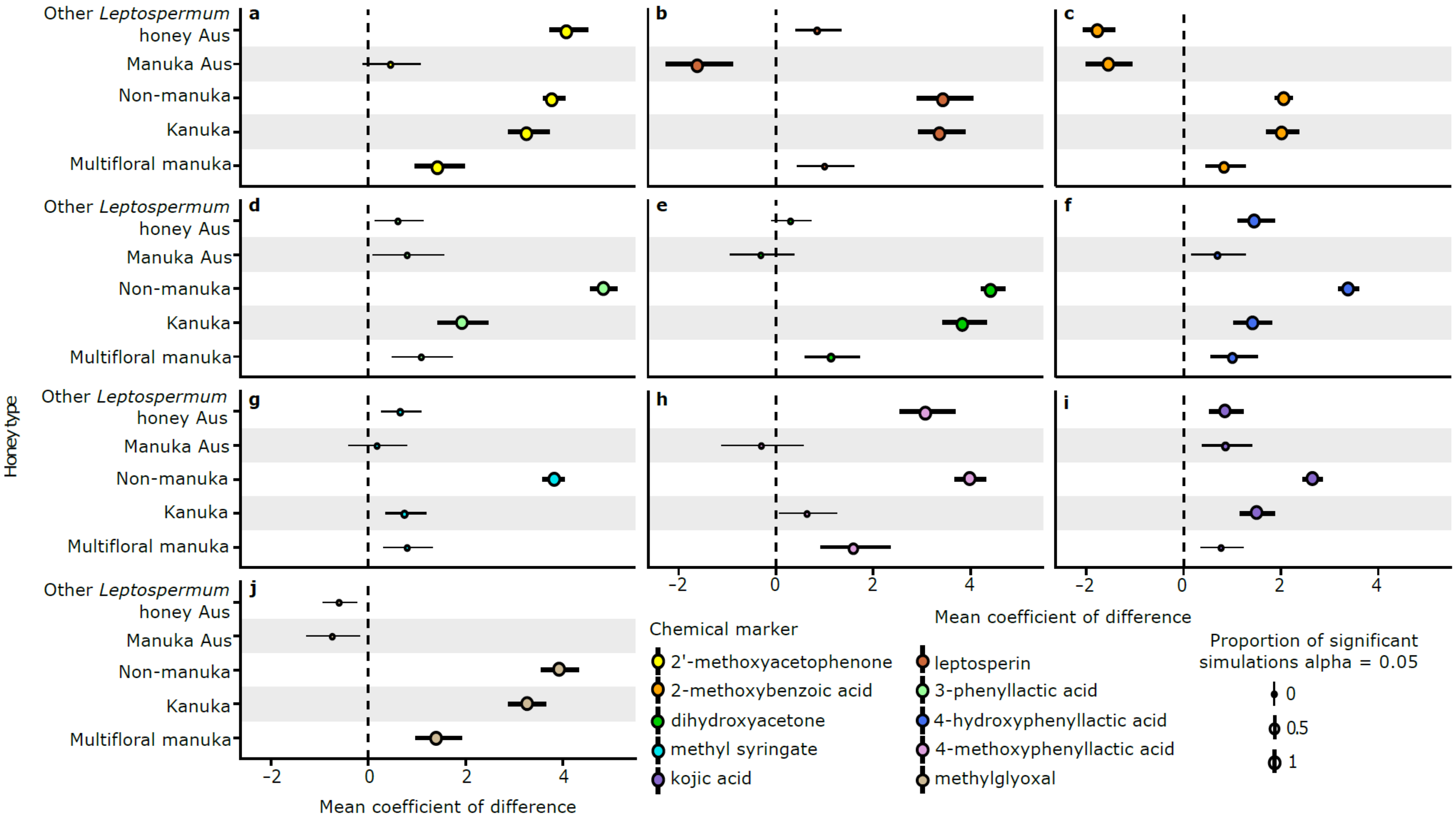

Supplementary Fig. 2 Bootstrap simulation results for concentration differences of each marker between monofloral manuka honey and the other honey types collected in 2014/15 from New Zealand and Australia. Error bars show the mean coefficient of difference ± 1 standard error of difference from the bootstrap simulations. (**a**) 2'-methoxyacetophenone (2'-MAP); (**b**) leptosperin; (**c**) 2-methoxybenzoic acid; (**d**) 3-phenyllactic acid; (**e**) dihydroxyacetone; (**f**) 4-hydroxyphenyllactic acid; (**g**) methyl syringate; (**h**) 4-methoxyphenyllactic acid; (**i**) kojic acid**;** (**j**) methylglyoxal.

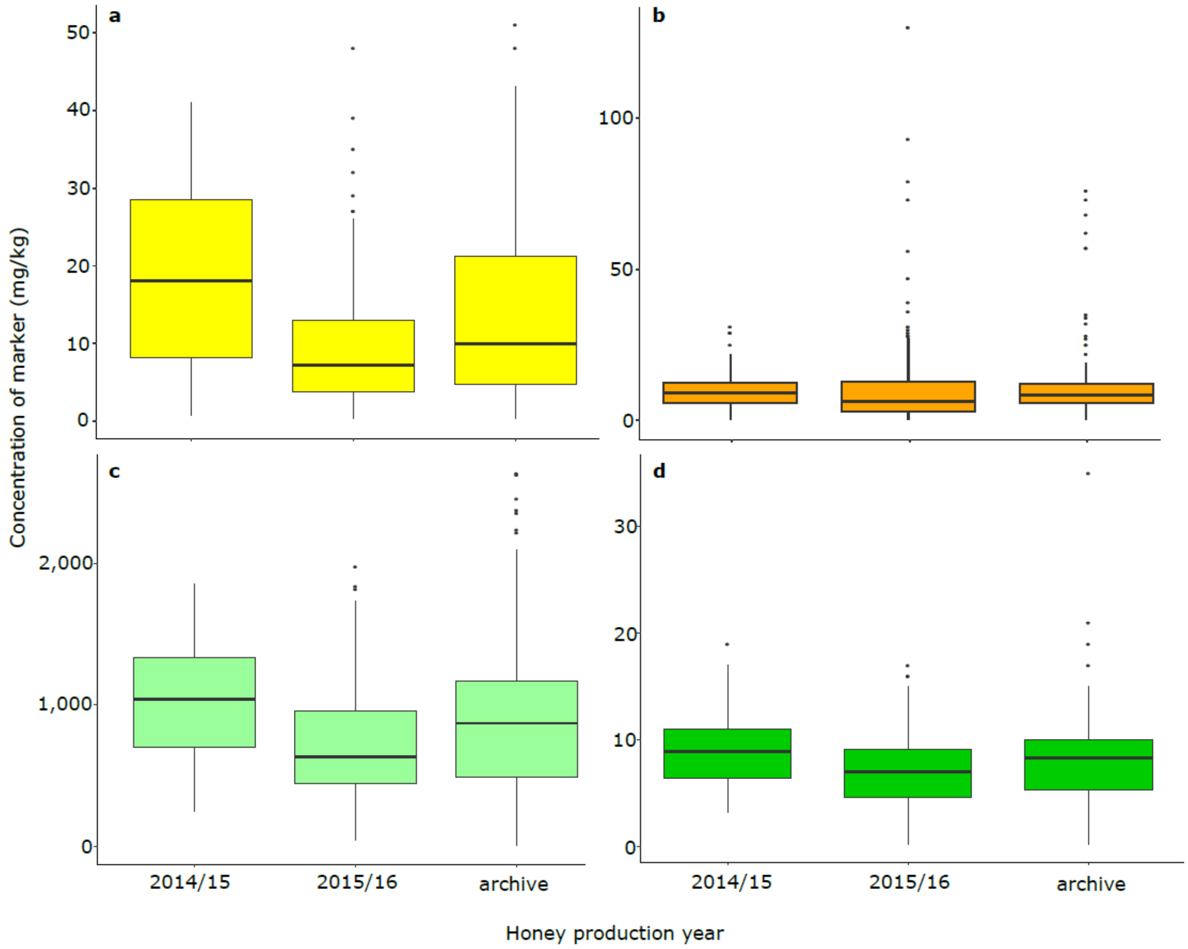

Supplementary Fig. 3 Concentration of four chemical markers in monofloral manuka honey collected in each year. Archive samples are honeys produced within the seven previous years which were stored until tested. (**a**). 2'-methoxyacetophenone; (**b**) 2-methoxybenzoic acid; (**c**) 3-pheyllactic acid; (**d**) 4-hydroxyphenyllactic acid. Boxplot elements include: the central line, median; box limits, first and third quartiles, whiskers, 1.5x inter-quartile range; points, outlier data beyond the end of the whiskers.

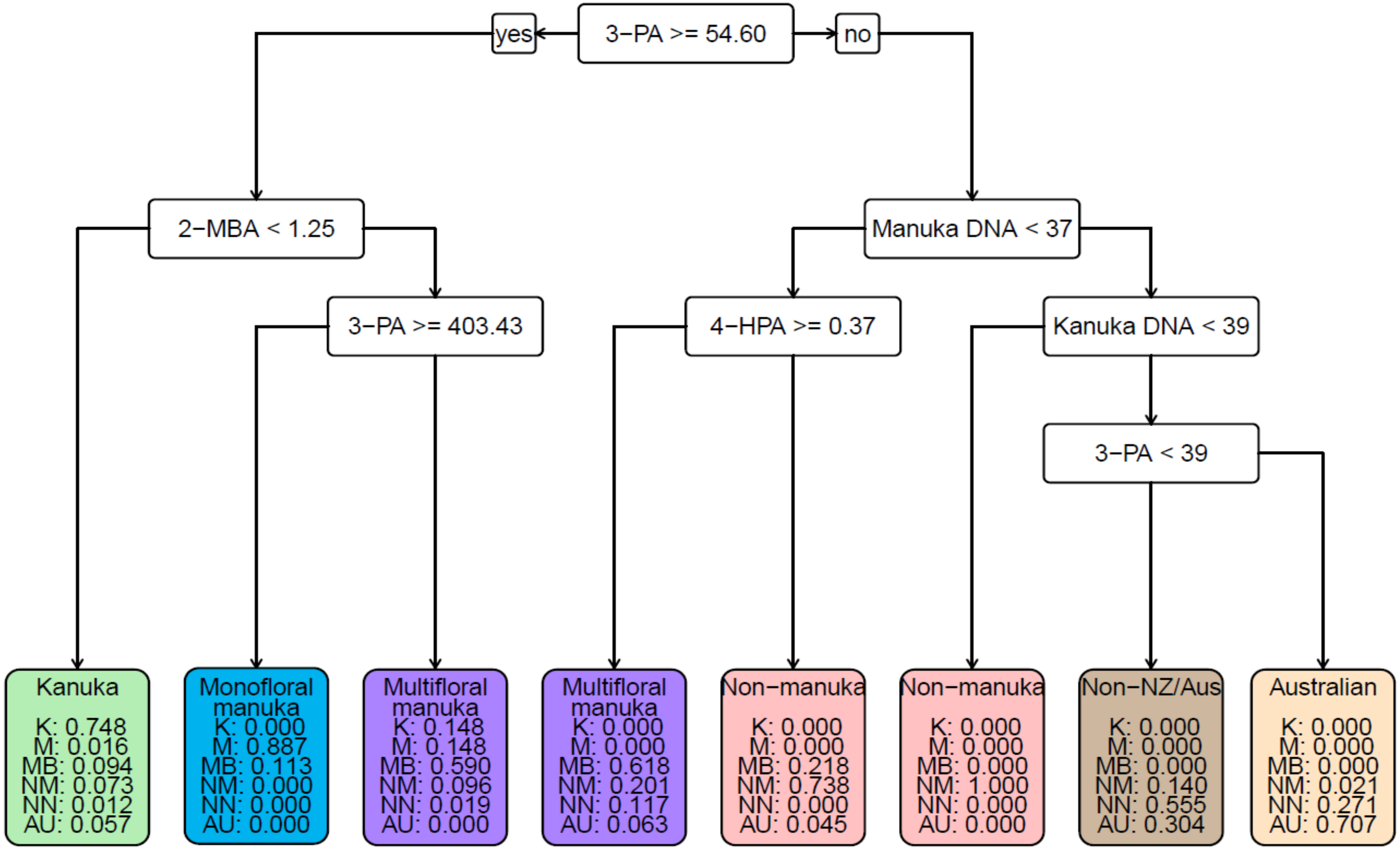

Supplementary Fig. 4 CART produced under the baseline scenario and built using 2014/15 data. At each split point in the tree, if the condition is met the path furthest left is chosen and if not then the path furthest right is chosen. The predicted class proportions at each terminal child node are shown. Class abbreviations are as follows: Kanuka honey (K); Monofloral manuka (M); Multifloral manuka (MB); Non-manuka (NM); None-NZ/Aus (NN) and Australian (AU). Units for chemical markers are mg/kg. Units for DNA markers are C_q.

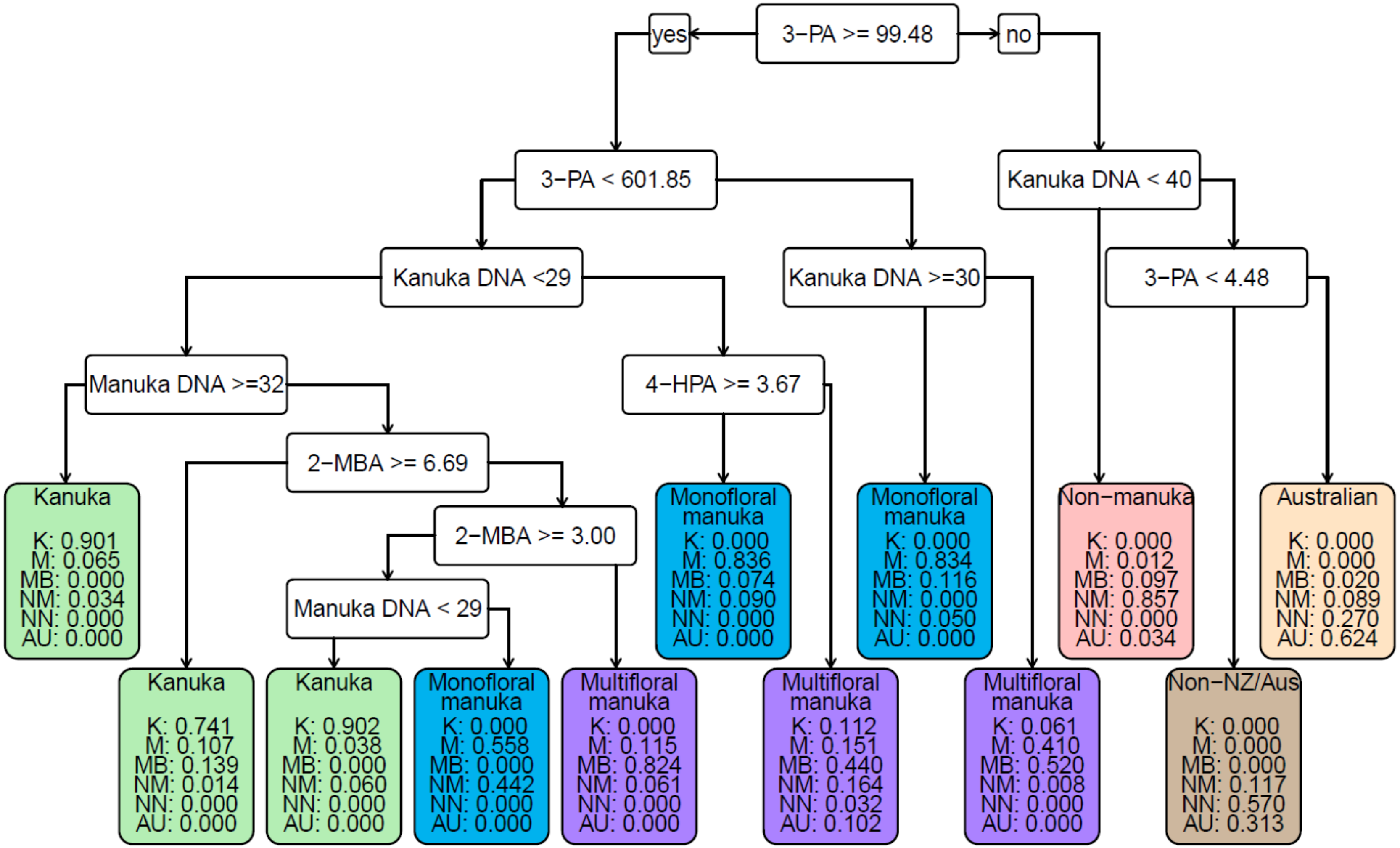

Supplementary Fig. 5 CART produced under the baseline scenario and built using 2015/16 data. At each split point in the tree, if the condition is met the path furthest left is chosen and if not then the path furthest right is chosen. The predicted class proportions at each terminal child node are shown. Class abbreviations are as follows: Kanuka honey (K); Monofloral manuka (M); Multifloral manuka (MB); Non-manuka (NM); None-NZ/Aus (NN) and Australian (AU). Units for chemical markers are mg/kg. Units for DNA markers are C_q.

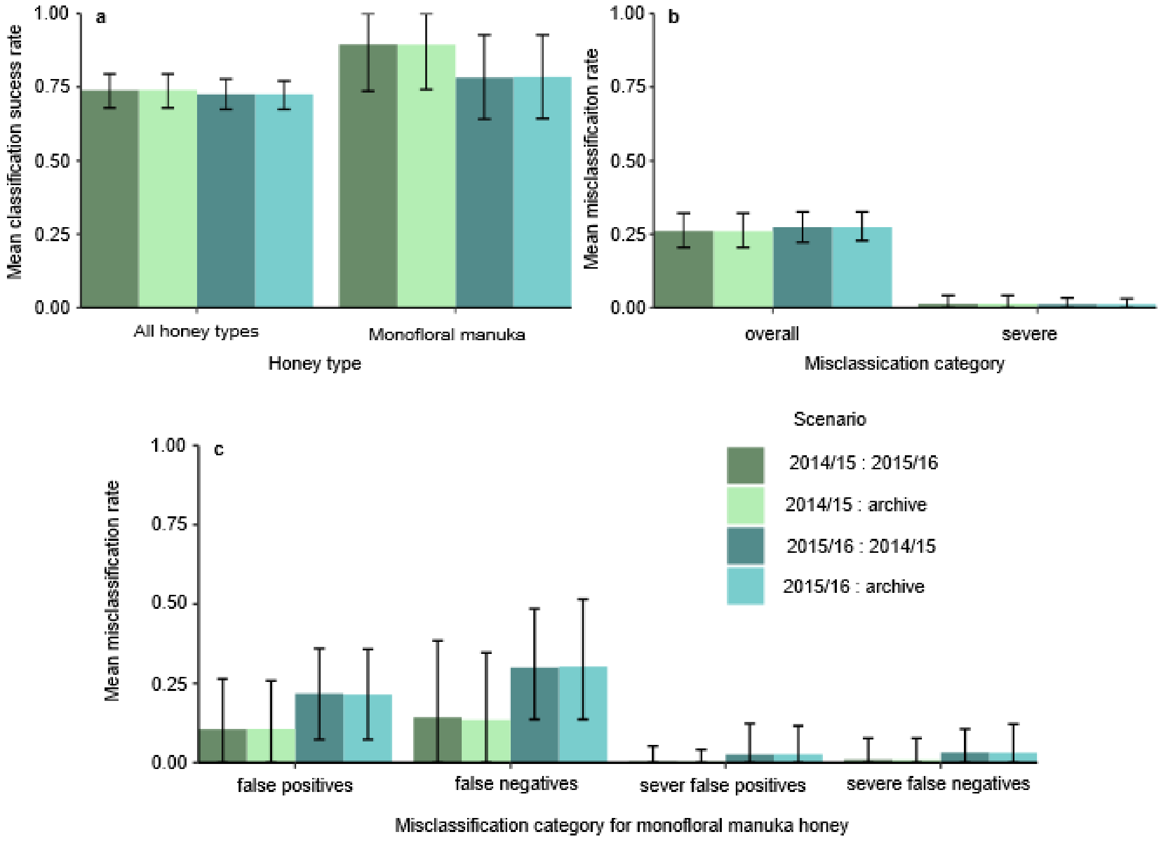

Supplementary Fig. 6 CART results for models built and tested using different honey production years: (a) Classification success for all honey types considered together and for monofloral manuka honey separately; (**b**) Overall and severe misclassification rates; (**c**) False positive, false negatives, severe false positives and severe false negatives for monofloral manuka honey. In the legend, the training set is before the colon and the test set is after the colon for both scenarios. Error bars represent the 95% confidence interval from the bootstrap distribution.

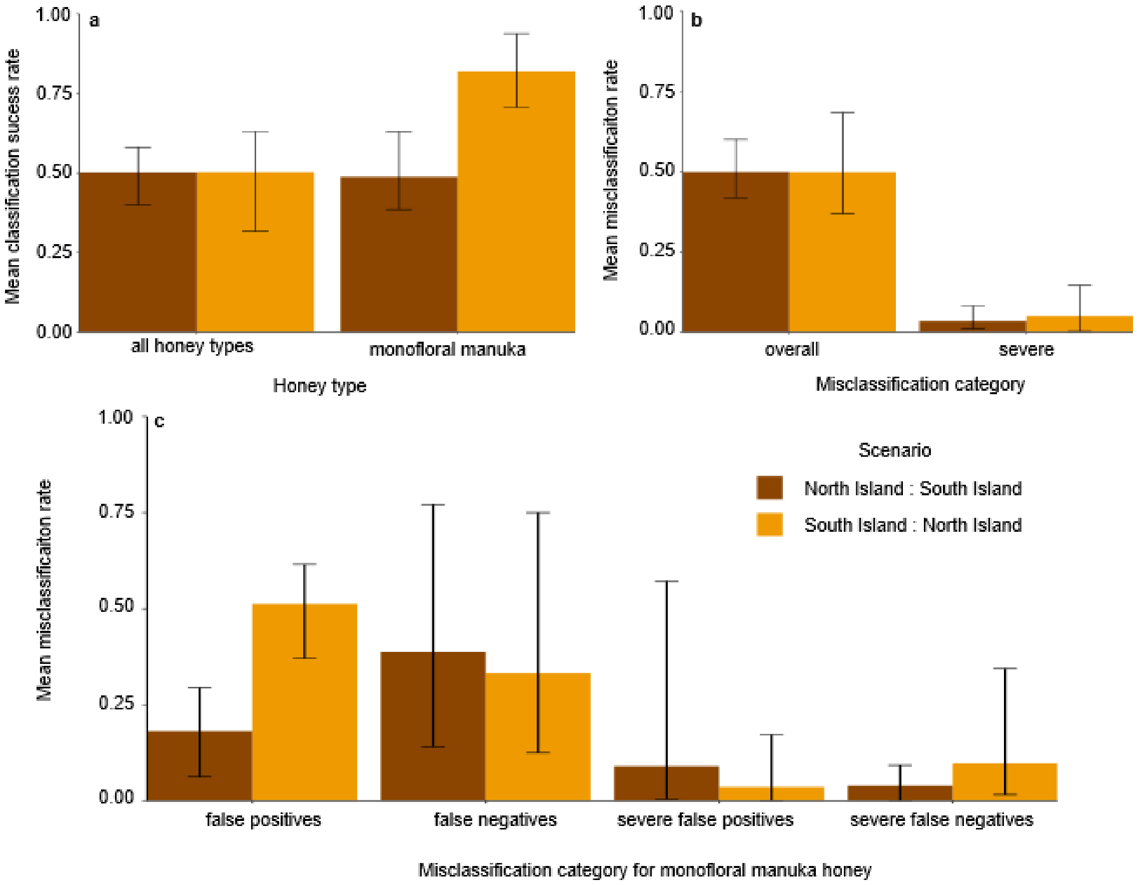

Supplementary Fig. 7 CART results for models built and tested using different honey production areas: (**a**) Classification success for all honey types considered together and for monofloral manuka honey separately; (**b**) Overall and severe misclassification rates; (**c**) False positive, false negatives, severe false positives and severe false negatives for monofloral manuka honey. In the legend, the training set is before the colon and the test set is after the colon for both scenarios. Error bars represent the 95% confidence interval from the bootstrap distribution

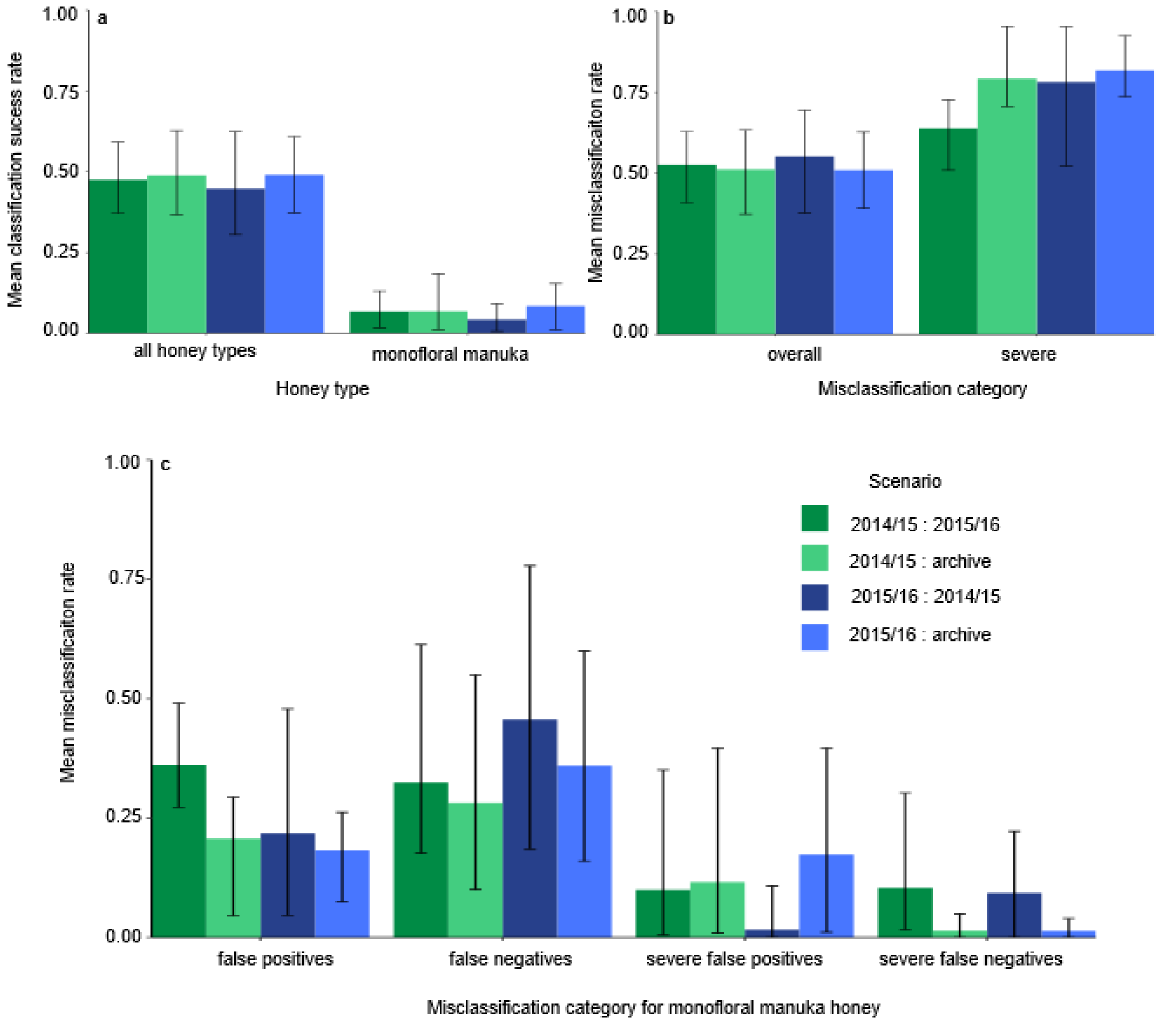

Supplementary Fig. 8 CART results for models built and tested using different honey production years with honey type as a 4-level response variable: (**a**) Classification success for all honey types considered together and for monofloral manuka honey separately; (**b**) Overall and severe misclassification rate; (**c**) False positive, false negatives, severe false positives and severe false negatives for monofloral manuka honey. For every scenario, the training set is before the colon and the test set is after the colon. Error bars represent the 95% confidence interval from the bootstrap distribution.

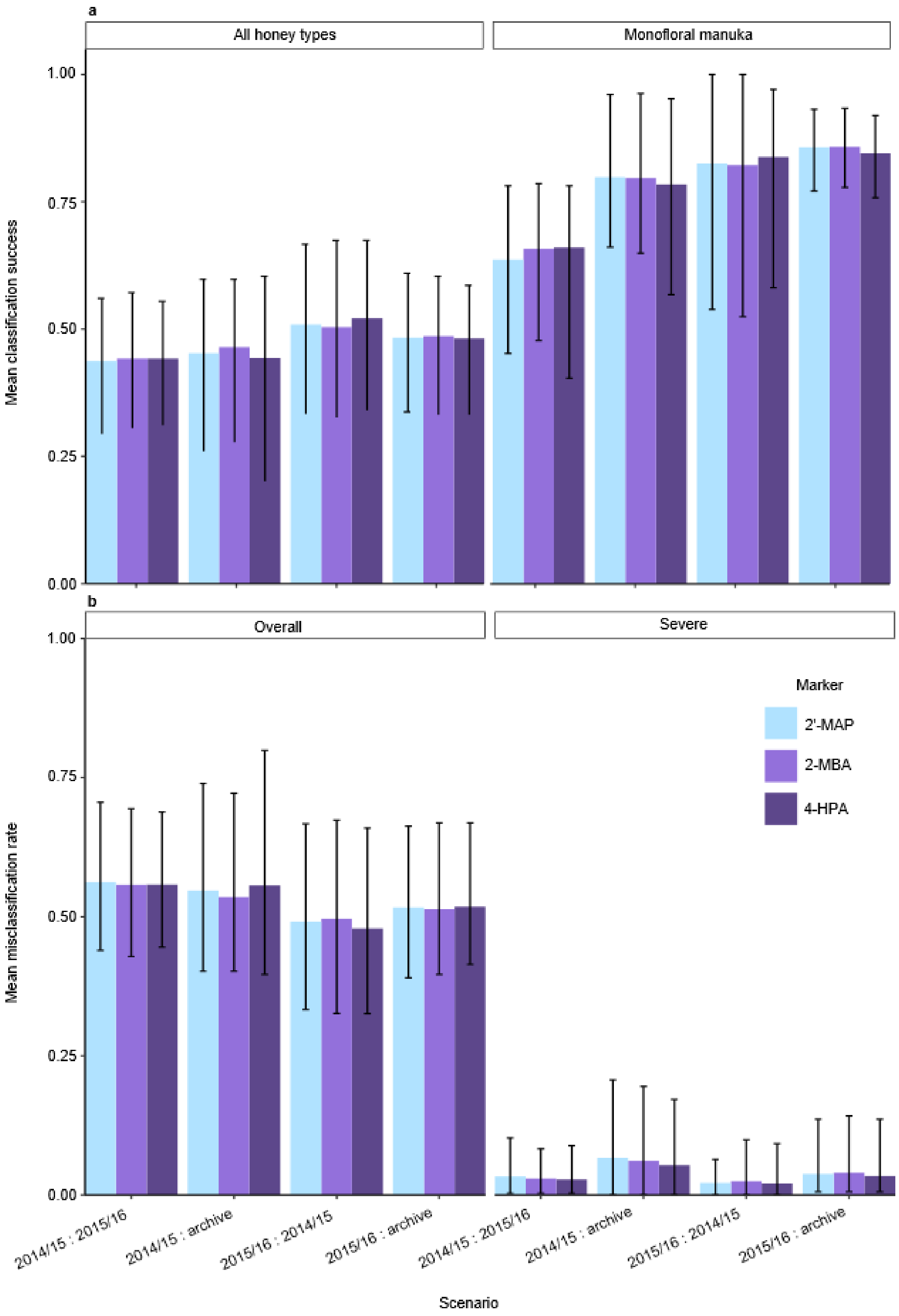

Supplementary Fig. 9 CART results for models built and tested using 3 different groups of markers as explanatory variables: 2'-MAP – model includes 3-PA, 2'-MAP, manuka and kanuka DNA; 2-MBA – model includes 3-PA, 2-MBA, manuka and kanuka DNA; 4-HPA – model includes 3-PA, 4-HPA, manuka and kanuka DNA. (**a**) Classification success for all honey types together and for monofloral manuka honey separately; (**b**) Overall and severe misclassification rates. For every scenario, the training set is before the colon and the test set is after the colon. Error bars represent the 95% confidence interval from the bootstrap distribution.

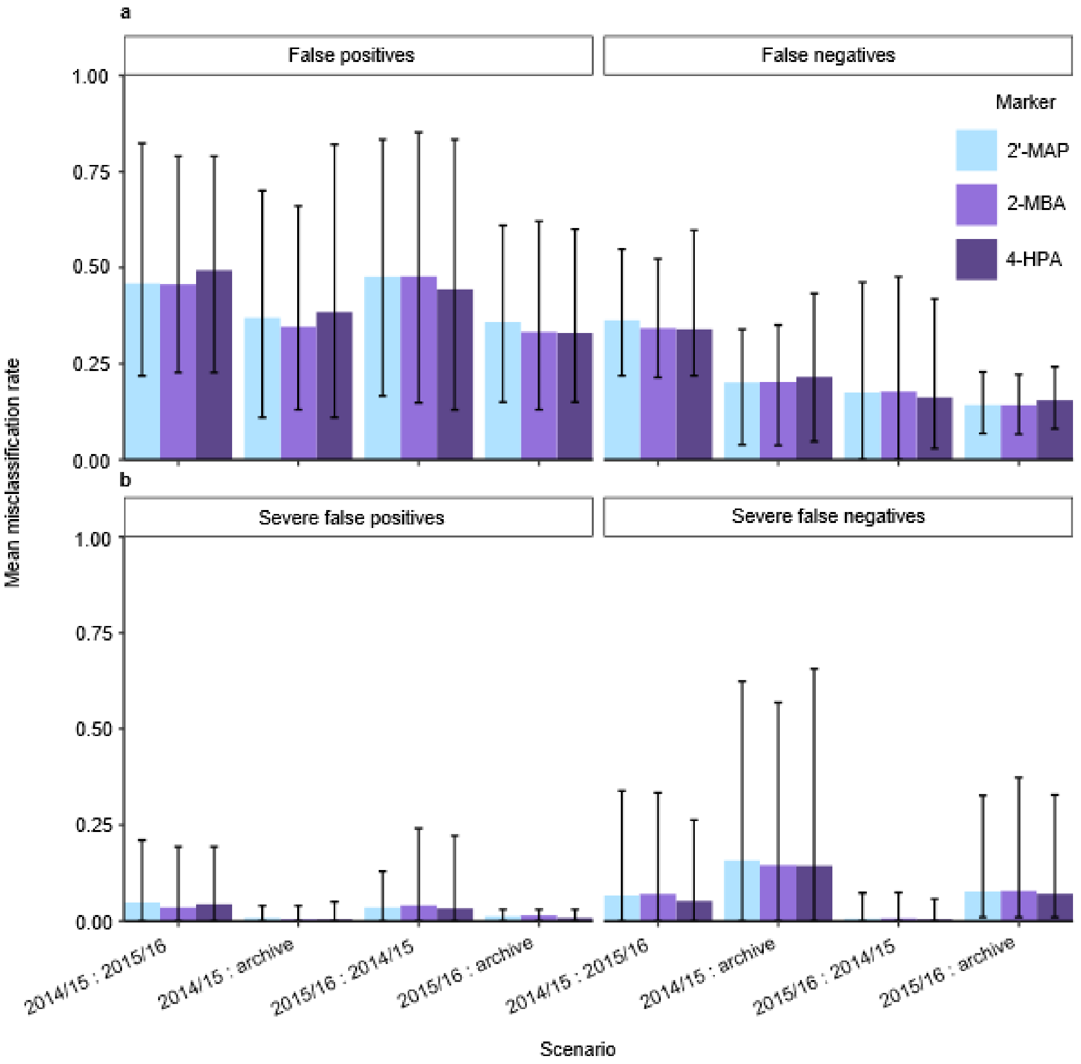

Supplementary Fig. 10 Misclassification category results for CARTs built and tested using 3 different groups of markers as explanatory variables: 2'-MAP – model includes 3-PA, 2'-MAP, manuka and kanuka DNA; 2-MBA – model includes 3-PA, 2-MBA, manuka and kanuka DNA; 4-HPA – model includes 3-PA, 4-HPA, manuka and kanuka DNA. (**a**) False positive and false negatives for monofloral manuka honey only; (**b**) Severe false positives and severe false negatives are for monofloral manuka honey only. For every scenario, the training set is before the colon and the test set is after the colon. Error bars represent the 95% confidence interval from the bootstrap distribution.

Supplementary Table 1 F-test results for significance testing of storage temperature and testing data using two separate linear models

	Storage temperature		Testing date	
**Chemical marker**	***F* value (3,15)**	***p* value**	***F* value (1,5)**	***p* value**
4-HPA	1.25	0.33	1.67	0.253
2-MBA	3.87	**0.03**	0.24	0.648
3-PA	5.81	**0.01**	4.20	0.100
Leptosperin	44.64	**<0.001**	35.71	**0.002**
MS	57.13	**<0.001**	26.15	**0.004**
2'-MAP	1.69	0.211	1.44	0.285
DHA	215.65	**<0.001**	191.63	**<0.001**
MG	30.43	**<0.001**	41.01	**0.001**

Significance at the α = 0.05 are in bold.

Supplementary Table 2 Linear model outputs for significant results of the changes in chemical marker with storage temperature

Chemical marker	Parameter	Coefficient Estimate	Std. Error	*t*-value (15)	*p*-value
3-PA	Intercept	5.68	0.01	739.52	<0.001
	4ºC	-0.01	0.01	0.91	0.379
	20ºC	-0.02	0.01	2.15	0.049
	35ºC	-0.03	0.01	3.94	0.001
2-MBA	Intercept	1.41	0.02	64.25	<0.001
	4ºC	-0.27	0.02	1.33	0.21
	20ºC	-0.04	0.02	1.78	0.10
	35ºC	0.03	0.02	1.30	0.21
Leptosperin	Intercept	5.95	0.01	422.39	<0.001
	4ºC	-0.02	0.01	1.14	0.272
	20ºC	-0.02	0.01	1.65	0.120
	35ºC	-0.14	0.01	10.28	<0.001
MS	Intercept	3.85	0.02	162.54	<0.001
	4ºC	-0.01	0.02	0.64	0.530
	20ºC	0.03	0.02	1.23	0.240
	35ºC	0.24	0.02	10.77	<0.001
DHA	Intercept	6.76	0.03	240.32	<0.001
	4ºC	-0.02	0.03	0.83	0.417
	20ºC	-0.11	0.03	4.11	0.001
	35ºC	-0.59	0.03	22.11	<0.001
MG	Intercept	5.21	0.05	112.55	<0.001
	4ºC	0.025	0.04	0.56	0.582
	20ºC	0.16	0.04	3.81	0.002
	35ºC	0.37	0.04	8.50	<0.001

Models are of the form log(marker) = intercept +b*$Sample_i$ + c*storage $temperature_j$. Only coefficient estimates for storage temperature are presented.

Supplementary Table 3 Linear model outputs for significant results of the changes in chemical marker with testing date

Chemical marker	Parameter	Coefficient Estimate	Std. Error	*t*-value (5)	*P* -value
Leptosperin	Intercept	5.96	0.01	478.96	<0.001
	Day 68	-0.06	0.01	5.98	0.002
MS	Intercept	3.85	0.02	163.12	<0.001
	Day 68	0.09	0.01	5.11	0.004
DHA	Intercept	6.75	0.02	337.02	<0.001
	Day 68	-0.21	0.06	13.84	<0.001
MG	Intercept	5.26	0.04	126.56	<0.001
	Day 68	0.12	0.03	6.40	0.001

Models are of the form log(marker) = intercept +b*Sample$_i$ + c*testing date$_j$ Only coefficient estimates for testing date are presented.

Supplementary Table 4 Analysis of variance summaries for assessing regional differences in chemical markers in *L. scoparium* nectar and manuka honey.

	***L. scoparium* nectar**		**Manuka honey**	
**Marker**	14/15 flowering season[1] (DoF_{num}=6,DoF_{den}=30)	15/16 flowering season[2] (DoF_{num}=11,DoF_{den}=103)	14/15 production year[3] (DoF_{num}=8,DoF_{den}=45)	15/16 production year[2] (DoF_{num}=11,DoF_{den}=204)
2'-MAP	F = 1.80<br>p = 0.13	F = 0.44<br>p = 0.94	**F = 5.27**<br>**p < 0.001**	F = 1.57<br>p = 0.11
4-HPA	F = 1.66<br>p = 0.16	F = 1.09<br>p = 0.38	**F = 3.79**<br>**p = 0.002**	F = 1.04<br>p = 0.41
2-MBA	F = 1.94<br>p = 0.11	F = 0.57<br>p = 0.85	F = 1.94<br>p =0.08	**F = 12.23**<br>**p < 0.001**
3-PA	**F = 12.23**<br>**p < 0.001**	F = 0.53<br>p = 0.876	**F = 3.19**<br>**p = 0.01**	**F = 5.48**<br>**p < 0.001**
MS	**F = 3.61**<br>**p = 0.01**	F = 0.69<br>p = 0.75	**F = 1.56**<br>**p = 0.16**	**F = 7.70**<br>**p < 0.001**
DHA	**F = 4.30**<br>**p < 0.001**	**F = 2.72**<br>**p = 0.004**	**F = 10.81**<br>**p < 0.001**	**F = 5.87**<br>**p < 0.001**
Leptosperin	**F = 8.16**<br>**p < 0.001**	Not tested	**F = 4.19**<br>**p = 0.001**	Not tested
MG	NA	NA	**F = 10.03**<br>**p < 0.001**	**F = 6.54**<br>**p < 0.001**

[1]Regions evaluated include: North Auckland, South Auckland, Gisborne, Wellington, Marlborough/Nelson, Canterbury and Otago.
[2]Regions evaluated include: North Auckland, South Auckland, Gisborne, Hawkes Bay, Taranaki, Wellington, Marlborough, Nelson, Canterbury, Westland, Otago and Southland,
[3]Regions evaluated include: North Auckland, South Auckland, Gisborne, Taranaki, Wellington, Marlborough, Nelson, Westland and Otago.
Significance at alpha = 0.05 are in bold.

Supplementary Table 5 Analysis of variance summaries for assessment of the mean difference between honey production years in the concentration of markers in manuka honey

Marker	2014/15 vs 2015/16	2014/15 vs archive	2015/2016 vs archive	Overall effect of season
2'-MAP	**t (268) = 4.541**<br>**p < 0.001**	**t (268) = 2.808**<br>**p = 0.01**	**t (268) = 2.025**<br>**p = 0.04**	**$F_{(2,268)}$ = 10.374**<br>**p < 0.001**
2-MBA	t (268) = 1.794<br>p = 0.111	t (268) = 0.087<br>p = 0.930	t (268) = 2.088<br>p = 0.111	$F_{(2,268)}$ = 2.78<br>p = 0.060
3-PA	**t (268) = 3.726**<br>**p < 0.001**	t (268) = 1.833<br>p = 0.067	**t (268) = 2.255**<br>**p = 0.02**	**$F_{(2,268)}$ = 7.38**<br>**p < 0.001**
4-HPA	**t (268) = 3.495**<br>**p = 0.002**	t (268) = 2.051<br>p = 0.062	t (268) = 1.698<br>p = 0.091	**$F_{(2,268)}$ = 6.199**<br>**p < 0.001**
MS	**t (268) = 2.545**<br>**p = 0.035**	**t (268) = 2.168**<br>**p = 0.047**	t (268) = 0.386<br>p = 0.700	**$F_{(2,268)}$ = 3.422**<br>**p = 0.034**
DHA	**t (223) = 1.364**<br>**p = 0.311**	t (223) = 0.417<br>p = 0.677	**t (223) = 2.422**<br>**p = 0.050**	**$F_{(2,223)}$ = 3.328**<br>**p = 0.040**
MGO	**t (223) = 1.163**<br>**p = 0.369**	t (223) = 0.049<br>p = 0.961	**t (223) = 3.192**<br>**p = 0.005**	**$F_{(2,223)}$ = 5.3159**<br>**p = 0.006**

Season is a 3-level factor consisting of 2014/15, 2015/16 and archive samples. Significance at alpha = 0.05 are in bold.

Supplementary Table 6 Summary of markers selected as explanatory variables across CART models built using samples from different honey production years, for both 6 and 4-level honey type response variables (2014/2015 and 2015/2016)

Marker	**Mean % of simulations where marker is selected as first split in tree** (min – max)		**Mean % of simulations where marker is selected at any point in the classification tree** (min – max)	
	**6-level**	**4-level**	**6-level**	**4-level**
**Kanuka DNA**	**21.73**<br>(9.10 – 35.0)	**0.60**<br>(0 – 1.40)	**92.18**<br>(84.40 – 100)	**81.12**<br>(62.10 – 98.60)
**Manuka DNA**	**13.38**<br>(11.80 – 14.60)	**48.68**<br>(29.70 – 68.50)	**92.13**<br>(84.50 – 99.60)	**97.90**<br>(95.80 – 100)
**2-MAP**	**1.80**<br>(0 – 4.00)	**11.33**<br>(6.20 – 16.80)	**50.30**<br>(22.80 – 78.00)	**51.25**<br>(30.80 – 71.00)
**2-MBA**	**0.53**<br>(0 – 1.30)	**1.08**<br>(0 – 2.40)	**53.65**<br>(46.30 – 60.40)	**50.25**<br>(20.30 – 79.70)
**3-PA**	**59.15**<br>(41.10 – 76.40)	**35.40**<br>(24.30 – 46.40)	**99.95**<br>(99.90 – 100)	**92.05**<br>(87.30 – 96.10)
**4-HPA**	**3.40**<br>(0 – 7.20)	**2.93**<br>(0 – 6.40)	**84.78**<br>(82.70 – 86.60)	**77.85**<br>(75.40 – 80.10)

Percentages are calculated from 1000 bootstrap simulations of each scenario.

Supplementary Table 7 Summary of markers selected as explanatory variables across CART models built using samples from different production areas (North Island (NI) and South Island (SI), New Zealand).

	NI training set and SI test set		SI training set and NI test set	
**Marker**	**% of simulations where variable is selected as first split in tree**	**% of simulations where variable is selected in the classification tree**	**% of simulations where variable is selected as first split in tree**	**% of simulations where variable is selected in the classification tree**
**Kanuka DNA**	0.00	98.10	14.50	93.70
**Manuka DNA**	0.00	98.20	9.40	98.00
**2-MAP**	0.10	90.90	0	87.20
**2-MBA**	0.30	84.00	0	77.10
**3-PA**	99.60	1.00	74.60	1.00
**4-HPA**	0.00	94.80	1.50	74.00

Percentages are calculated from 1000 bootstrap simulations of each scenario.

Supplementary Table 8 Summary of markers selected as explanatory variables when 3 different groups of markers are used.

	**Mean % of simulations where marker is selected as first split in tree** (min – max)			**Mean % of simulations where marker is selected at any point in the classification tree** (min – max)		
**Marker**	**2-MAP**	**2-MBA**	**4-HPA**	**2-MAP**	**2-MBA**	**4-HPA**
**Kanuka DNA**	**22.17** (8.20 – 36.70)	**23.50** (9.50 – 39.10)	**21.98** (8.90 – 35.40)	**97.35** (94.30 – 99.90)	**93.18** (85.50 – 100)	**99.53** (98.60 – 100)
**Manuka DNA**	**13.68** (13.20 – 14.40)	**14.40** (13.40 – 17.20)	**14.25** (12.50 – 16.20)	**95.20** (90.20 – 100)	**96.78** (93.60 – 99.90)	**96.63** (93.1 – 100)
**3-PA**	**61.83** (44.50 – 78.90)	**61.63** (46.30 – 77.10)	**60.05** (43.50 – 78.60)	**100** (100 – 100)	**100** (100 – 100)	**99.98** (99.90 – 100)
**2-MAP**	**2.33** (0 – 4.90)	NA	NA	**73.05** (56.00 – 89.70)	NA	NA
**2-MBA**	NA	**0.48** (0 – 1.10)	NA	NA	**74.23** (63.10 – 82.90)	NA
**4-HPA**	NA	NA	**3.73** (0 – 7.60)	NA	NA	**90.88** (86.60 – 94.60)

This was tested using CARTs built with samples from different honey production years (2014/2015 and 2015/2016). 2’-MAP – model includes 3-PA, 2’-MAP, manuka and Kanuka DNA; 2-MBA – model includes 3-PA, 2-MBA, manuka and Kanuka DNA; 4-HPA – model includes 3-PA, 4-HPA, manuka and kanuka DNA. Percentages are calculated from 1000 bootstrap simulations of each scenario.

Supplementary Table 9 Reclassification of honey samples from CARTs built using chemical marker data truncated at the LOR and with simulated LOR values

Training data	Test data	Honey type* predicted from CART using LOR values	Mean number of samples reclassified using simulated LOR values (% of simulations with reclassification)	Honey type of reclassified samples
2014/15	2015/16	Monofloral manuka	0.22 (0.5%)	Kanuka
		Multifloral manuka	0.11 (0.5%)	Kanuka
			4.82 (99.4%)	Non-manuka
		Kanuka	0.03 (0.5%)	Monofloral manuka
			1.60 (12.6%)	Multifloral manuka
		Non-manuka	17.84 (100%)	Multifloral manuka
		Australian	0.05 (4.1%)	Non-NZ/Aus
		Non-NZ/Aus	0.09 (2.6%)	Non-manuka
			1.93 (58.1%)	Australian
	archive	Monofloral manuka	0.17 (0.5%	Kanuka
		Multifloral manuka	0.048 (0.5%)	Kanuka
		Kanuka	0.02 (0.5%)	Monofloral manuka
			0.43 (12.5%)	Multifloral manuka
		Non-manuka	1.49 (73.5%)	Multifloral manuka
		Australian	0.22 (5.9%)	Non-NZ/Aus
		Non-NZ/Aus	0.05 (2.5%)	Non-manuka
			1.41 (53.0%)	Australian
2015/16	2014/15	Monofloral manuka	0.46 (7.7%)	Kanuka
		Multifloral manuka	0.07 (4.6%)	Monofloral manuka
			0.42 (7.7%)	Kanuka
			0.02 (2.2%)	Non-manuka
		Kanuka	0.09 (4.6%)	Monofloral manuka
			0.002 (0.2%)	Multifloral manuka
			0.006 (0.6%)	Non-manuka
		Australian	0.30 (4%)	Non-manuka
			0.32 (8.7%)	Non-NZ/Aus
		Non-NZ/Aus	2.08 (36.2%)	Non-manuka
			2.07 (5.5%)	Australian
	archive	Monofloral manuka	0.03 (3.3%)	Kanuka
		Multifloral manuka	0.13 (4.6%)	Monofloral manuka
			0.59 (16.8%)	Kanuka
			0.07 (2.2%)	Non-manuka
		Kanuka	0.18 (4.6%)	Monofloral manuka
			0.02 (1.3%)	Multifloral manuka
			0.01 (1.1%)	Non-manuka
		Australian	0.004 (0.3%)	Non-manuka
			0.43 (10.2%)	Non-NZ/Aus
		Non-NZ/Aus	0.36 (34.5%)	Non-manuka
			1.52 (51.4%)	Australian

*Comparison of honey types where no reclassification occurred in the scenarios are not listed.

Supplementary Table 10 Reclassification of honey samples from CARTs built using raw and adjusted manuka and Kanuka DNA markers (values < LOR are changed to 40)

Training data	Test data	Honey type predicted from CART using raw Cq values	% of samples reclassified using adjusted Cq values	Honey type of reclassified samples
2014/15	2015/16	Non-manuka (*n* = 75)	9	Non-NZ/Aus
			5	Australian
	archive	Non-manuka (*n* = 8)	50	Australian
2015/16	2014/15	Non-manuka (*n* = 33)	18	Non-NZ/Aus
			3	Australian
		Australian (*n* = 15)	93	Non-manuka
		Non-NZ/Aus (*n* = 20)	25	Non-manuka
	archive	Non-manuka (n = 8)	38	Australian
		Australian (*n* = 16)	13	Non-manuka
		Non-NZ/Aus (*n* = 15)	7	Non-manuka

No samples were reclassified for Kanuka, monofloral manuka or multifloral manuka honey in any of the scenarios.

Supplementary Table 11 Percentage of honey samples from New Zealand from each test dataset that were reclassified after all the values of all 4 chemical markers were systematically increased or decreased to represent laboratory variation

		% of samples reclassified after downwards bias			% of samples reclassified after upwards bias		
**Definition**	**Test dataset**	**5%**	**10%**	**20%**	**5%**	**10%**	**20%**
Option 3	2014/2015	0.0	0.7	2.1	1.4	2.1	2.8
	2015/2016	1.7	3.7	7.4	0.6	2.9	3.7
	archive	6.5	2.4	1.2	0.0	1.8	4.1
Option 4	2014/2015	0.7	1.4	2.8	2.1	2.8	4.3
	2015/2016	2.0	5.4	9.7	0.8	2.9	4.9
	archive	1.2	2.4	7.7	1.8	1.8	4.7

Supplementary Table 12 Percentage of honey samples from New Zealand from each test dataset that were reclassified after all the values of both the manuka and Kanuka DNA markers were systematically increased or decreased to represent laboratory variation

		% of samples reclassified after downwards bias			% of samples reclassified after upwards bias		
**Definition**	**Test dataset**	**5%**	**10%**	**20%**	**5%**	**10%**	**20%**
Option 3	2014/2015	0.7	2.8	3.5	4.3	7.8	9.2
	2015/2016	3.6	6.6	5.4	2.6	4.6	15.7
	archive	1.8	5.9	11.2	4.1	6.5	27.8
Option 4	2014/2015	0.0	0.0	0.0	1.4	2.8	9.2
	2015/2016	0.0	0.3	0.3	0.9	2.7	18.0
	archive	1.8	3.6	8.9	4.1	7.7	29.0

Supplementary Table 13 Plant species collected for the study

Country	Species	Common name	Number
New Zealand	*Echium vulgare*	viper's bugloss	12
	Ixerba brexioides	tawari	15
	Knightia excelsa	rewarewa	10
	Kunzea ericoides/K. robusta	Kanuka	69
	K. sinclairii	Great Barrier Island Kanuka	3
	K. tenauculis	geothermal Kanuka	3
	Kunzea serotina	makahikatoa	6
	Kunzea spp. hybrid	*Kunzea* spp. hybrid	3
	Leptospermum scoparium[1]	manuka	152
	Leptospermum spp[2]	NA	35
	Metrosideros excelsa	pōhutukawa	25
	Metrosideros umbellata/M. robusta	northern and southern rātā	12
	Nothofagus solandri[3]	honeydew	20
	Thymus vulgaris	thyme	3
	Trifolium repens	white clover	38
	Ulex europaeus	common gorse	16
	Weinmannia racemosa	kāmahi	23
Australia	*L. grandifolium*	mountain tea tree	3
	L. laevigatum	coastal tea tree	8
	L. liversidgei	olive tea tree	9
	L. polygalifolium	Tantoon, yellow tea tree	10
	L. scoparium	manuka	12

[1] 2015/16 samples were collected from five different habitat types from each region, where possible.

[2]*Leptospermum* species not native, but collected in New Zealand include: *L. brachyandrum, L. grandifolium. L. lanigerum, L. morrisonii, L. myrtifolium, L. obovatum, L. petersonii, L. polygalifolium, L. rupestre* and two *Leptospermum spp.* not identified to species level.

[3]The *Nothofagus* genus has recently been split into four genera with two present in New Zealand: *Fuscospora* and *Lophozonia*. *N. solandri* has been assigned to *Fuscospora*. The sample collected from *Nothofagus solandri* is not a nectar sample but a sample of the liquid excreted from insects feeding on the phloem sap of the tree rather than the nectar taken from the flower.

Supplementary Table 14 Honey samples sourced and tested for the study

Honey type as identified by supplier	Number	Standardised honey type[1]
Bush	10	Non-manuka
Bush blend	18	Multifloral manuka
Clover	61	Non-manuka
Clover blend	15	Non-manuka
Honeydew	15	Non-manuka
Kāmahi	22	Non-manuka
Kanuka	21	Kanuka
Kunzea species	9	Kanuka
Monofloral manuka	273	Monofloral manuka
Multifloral manuka	74	Multifloral manuka
Manuka honeydew	3	Multifloral manuka
Multifloral	65	Non-manuka
Other monofloral	9	Non-manuka
Pōhutukawa	7	Non-manuka
Rātā	5	Non-manuka
Rewarewa	6	Non-manuka
Tawari	23	Non-manuka
Thyme	14	Non-manuka
Tussock grassland	3	Non-manuka
Viper's bugloss	3	Non-manuka
Willow honeydew	4	Non-manuka
Australia	64	Australian
Non-NZ/Aus[2]	71	Non-NZ/Aus

Note: 2014/15 samples = 141; 2015/16 samples = 350; archive samples = 169.
[1] Supplier descriptions were standardised using defined rules. If multiple floral sources were provided and one of them was manuka, it would be termed 'multifloral manuka'. Whereas, samples named with multiple floral sources, but with no reference to manuka, were termed 'multifloral'.
[2] Countries honey was collected from include: Botswana, Brazil, Canada, China, Germany, India, Italy, Mexico, Republic of South Africa, Swaziland, Tanzania (and Zanzibar), United Kingdom, United States of America, Vietnam and Zambia.
